# Clinicopathologic Heterogeneity and Glial Activation Patterns in Alzheimer Disease

**DOI:** 10.1001/jamaneurol.2024.0784

**Published:** 2024-04-15

**Authors:** Naomi Kouri, Isabelle Frankenhauser, Zhongwei Peng, Sydney A. Labuzan, Baayla D. C. Boon, Christina M. Moloney, Cyril Pottier, Daniel P. Wickland, Kelsey Caetano-Anolles, Nick Corriveau-Lecavalier, Jessica F. Tranovich, Ashley C. Wood, Kelly M. Hinkle, Sarah J. Lincoln, A. J. Spychalla, Matthew L. Senjem, Scott A. Przybelski, Erica Engelberg-Cook, Christopher G. Schwarz, Rain S. Kwan, Elizabeth R. Lesser, Julia E. Crook, Rickey E. Carter, Owen A. Ross, Christian Lachner, Nilüfer Ertekin-Taner, Tanis J. Ferman, Julie A. Fields, Mary M. Machulda, Vijay K. Ramanan, Aivi T. Nguyen, R. Ross Reichard, David T. Jones, Jonathan Graff-Radford, Bradley F. Boeve, David S. Knopman, Ronald C. Petersen, Clifford R. Jack, Kejal Kantarci, Gregory S. Day, Ranjan Duara, Neill R. Graff-Radford, Dennis W. Dickson, Val J. Lowe, Prashanthi Vemuri, Melissa E. Murray

**Affiliations:** 1 Department of Neuroscience, Mayo Clinic, Jacksonville, Florida; 2 Paracelsus Medical Private University, Salzburg, Austria; 3 Department of Quantitative Health Sciences, Mayo Clinic, Jacksonville, Florida; 4 Department of Radiology, Mayo Clinic, Rochester, Minnesota; 5 Department of Neurology, Mayo Clinic, Rochester, Minnesota; 6 Department of Quantitative Health Sciences, Mayo Clinic, Rochester, Minnesota; 7 Department of Psychiatry and Psychology, Mayo Clinic, Jacksonville, Florida; 8 Department of Neurology, Mayo Clinic, Jacksonville, Florida; 9 Department of Psychiatry and Psychology, Mayo Clinic, Rochester, Minnesota; 10 Department of Laboratory Medicine and Pathology, Mayo Clinic, Rochester, Minnesota; 11 Wien Center for Alzheimer’s Disease and Memory Disorders, Mount Sinai Medical Center, Miami Beach, Florida

## Abstract

**Importance:**

Factors associated with clinical heterogeneity in Alzheimer disease (AD) lay along a continuum hypothesized to associate with tangle distribution and are relevant for understanding glial activation considerations in therapeutic advancement.

**Objectives:**

To examine clinicopathologic and neuroimaging characteristics of disease heterogeneity in AD along a quantitative continuum using the corticolimbic index (CLix) to account for individuality of spatially distributed tangles found at autopsy.

**Design, Setting, and Participants:**

This cross-sectional study was a retrospective medical record review performed on the Florida Autopsied Multiethnic (FLAME) cohort accessioned from 1991 to 2020. Data were analyzed from December 2022 to December 2023. Structural magnetic resonance imaging (MRI) and tau positron emission tomography (PET) were evaluated in an independent neuroimaging group. The FLAME cohort includes 2809 autopsied individuals; included in this study were neuropathologically diagnosed AD cases (FLAME-AD). A digital pathology subgroup of FLAME-AD cases was derived for glial activation analyses.

**Main Outcomes and Measures:**

Clinicopathologic factors of heterogeneity that inform patient history and neuropathologic evaluation of AD; CLix score (lower, relative cortical predominance/hippocampal sparing vs higher, relative cortical sparing/limbic predominant cases); neuroimaging measures (ie, structural MRI and tau-PET).

**Results:**

Of the 2809 autopsied individuals in the FLAME cohort, 1361 neuropathologically diagnosed AD cases were evaluated. A digital pathology subgroup included 60 FLAME-AD cases. The independent neuroimaging group included 93 cases. Among the 1361 FLAME-AD cases, 633 were male (47%; median [range] age at death, 81 [54-96] years) and 728 were female (53%; median [range] age at death, 81 [53-102] years). A younger symptomatic onset (Spearman ρ = 0.39, *P* < .001) and faster decline on the Mini-Mental State Examination (Spearman ρ = 0.27; *P* < .001) correlated with a lower CLix score in FLAME-AD series. Cases with a nonamnestic syndrome had lower CLix scores (median [IQR], 13 [9-18]) vs not (median [IQR], 21 [15-27]; *P* < .001). Hippocampal MRI volume (Spearman ρ = −0.45; *P* < .001) and flortaucipir tau-PET uptake in posterior cingulate and precuneus cortex (Spearman ρ = −0.74; *P* < .001) inversely correlated with CLix score. Although AD cases with a CLix score less than 10 had higher cortical tangle count, we found lower percentage of CD68-activated microglia/macrophage burden (median [IQR], 0.46% [0.32%-0.75%]) compared with cases with a CLix score of 10 to 30 (median [IQR], 0.75% [0.51%-0.98%]) and on par with a CLix score of 30 or greater (median [IQR], 0.40% [0.32%-0.57%]; *P* = .02).

**Conclusions and Relevance:**

Findings show that AD heterogeneity exists along a continuum of corticolimbic tangle distribution. Reduced CD68 burden may signify an underappreciated association between tau accumulation and microglia/macrophages activation that should be considered in personalized therapy for immune dysregulation.

## Key Points

**Question:**

How is corticolimbic vulnerability to tau pathology as a continuous trait associated with clinicopathologic heterogeneity and glial activation patterns in neuropathologically diagnosed Alzheimer disease (AD)?

**Findings:**

In this cross-sectional study including 1361 neuropathologically diagnosed AD cases, clinicopathologic variables ranked as highly important to corticolimbic vulnerability were age at symptomatic onset, disease duration, Braak stage, and nonamnestic clinical syndrome. AD cases with relative cortical predominance/hippocampal sparing exhibited higher cortical tau pathology but diminished levels of activated microglia/macrophages.

**Meaning:**

These results suggest that quantitative capture of corticolimbic vulnerability was associated with a constellation of clinicopathologic factors that were highly associated with age at symptomatic onset and highlight an altered cortical microglia/macrophage response in AD.

## Teaser

This cross-sectional study evaluates the association between corticolimbic vulnerability to tau pathology as a continuous trait with clinicalpathologic heterogeneity and glial activation patterns in neuropathologically diagnosed Alzheimer disease.

## Introduction

Neuropathologic examination of an Alzheimer disease (AD) brain provides the foundational science from which we may better understand the topographic landscape underlying heterogeneity of nonamnestic and amnestic clinical syndromes.^1,2,3,4,5^ As the leading cause of dementia in older adults, uncovering neuropathologic underpinnings of clinicopathologic heterogeneity in AD remains critical to inform biomarker interpretation and patient care.^6^ Selective corticolimbic vulnerability in AD inspired a series of studies investigating neurofibrillary tangle distributions to objectively classify 3 AD neuropathologic subtypes: hippocampal sparing with relative cortical predominance, typical/representative, and limbic predominant with relative cortical sparing.^2,3,7,8,9,10^ We and others found striking demographic and clinical differences among these AD subtypes including sex, age at symptomatic onset, nonamnestic clinical syndrome, rate of cognitive decline, and cholinergic hub vulnerability.^2,3,7,10,11,12^ To expand our understanding of clinicopathologic heterogeneity in AD, we designed an innovative approach to both quantify and classify a corticolimbic index (CLix) of relational tangle distribution as a continuous trait. The Florida Autopsy Multiethnic series^13^ (FLAME-AD) was investigated for the importance of clinicopathologic factors in predicting the CLix score of tangle distribution using random forest regression modeling. An independent neuroimaging group was used to visualize the association between structural magnetic resonance imaging (MRI) and tau positron emission tomography (tau-PET) with the neuropathologically defined CLix score.

As gliosis plays a fundamental role in AD pathogenesis,^14,15,16,17,18^ we evaluated 2 robust glial markers in the human brain across 5 corticolimbic brain regions to compare patterns with tau (phosphorylation-dependent anti-tau antibody 8 [AT8], anti-tau AD antibody [GT-38]) and amyloid-β (6F/3D). Astrogliosis was measured using glial fibrillary acidic protein (GFAP), an intermediate filament protein highly expressed by reactive astrocytes.^19^ Activated microglia/macrophages were measured using CD68, a glycoprotein highly expressed in the lysosomes of activated myeloid cells.^20,21^ We hypothesized that clinicopathologic heterogeneity measures and glial activation markers would differ among CLix-classified AD subtypes and provide further neurobiologic insight into selective vulnerability observed in AD brains. Thus, in the context of corticolimbic tangle distribution our goals were to (1) evaluate the importance of demographic and clinical measures relevant to a patient’s medical history, (2) assess the association of antemortem MRI with tau-PET measures, and (3) use digital pathology to evaluate glial activation patterns.

## Methods

### Participants

**Figure 1.**
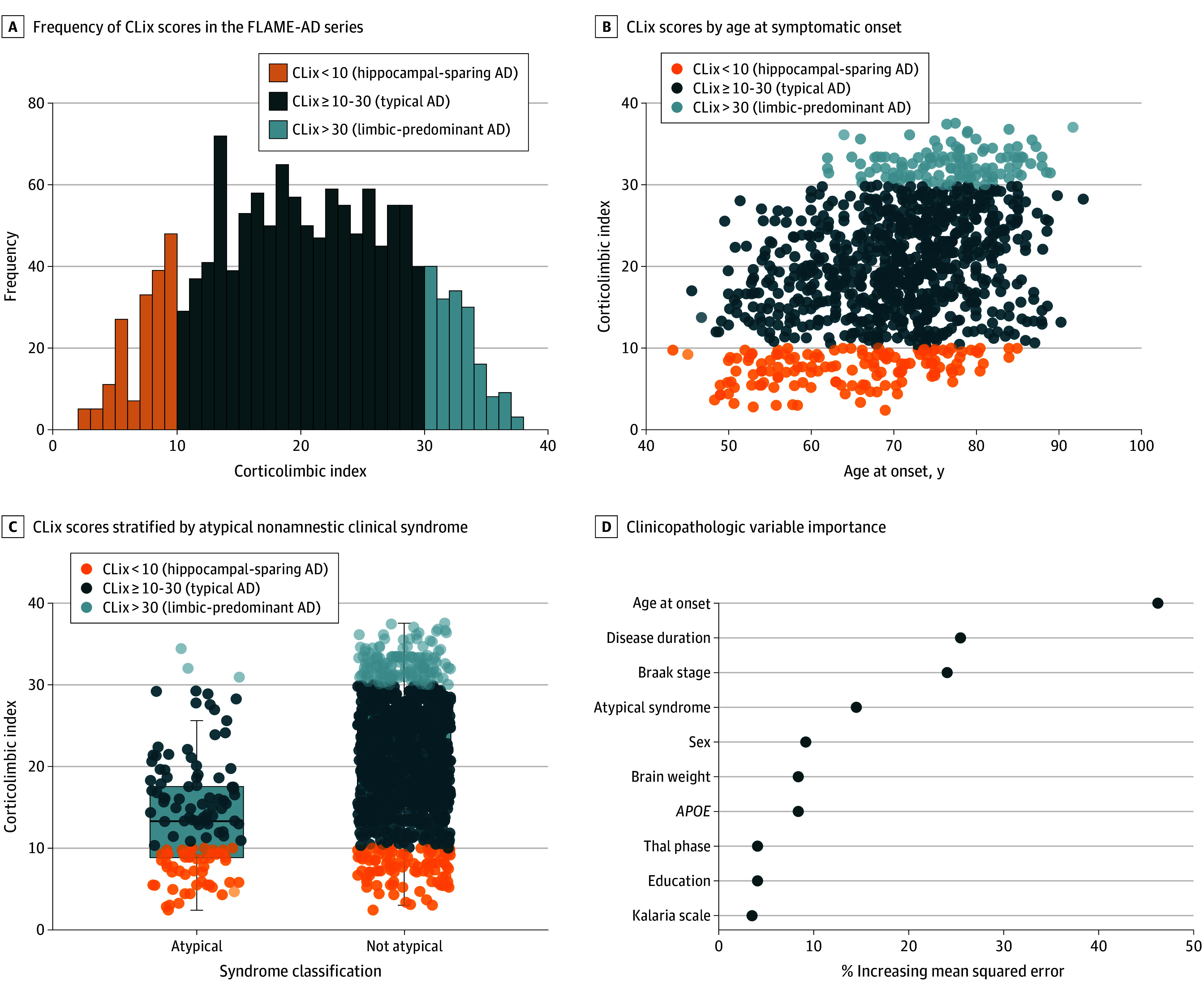
Clinicopathologic Heterogeneity in Alzheimer Disease (AD) Corticolimbic index (CLix) quantitatively defines corticolimbic vulnerability as a continuous measure (range, 0-40) calculated from the means and proportions of thioflavin-S–positive neurofibrillary tangle counts from hippocampus (CA1 and subiculum) and association cortices (superior temporal, inferior parietal, and middle frontal). A, The frequency of CLix scores in the Florida Autopsied Multiethnic (FLAME-AD) series. B, CLix scores by age at symptomatic onset. C, CLix scores stratified by atypical, nonamnestic clinical syndrome. D, Lolliplot of clinicopathologic variable importance from random forest regression model.

**Figure 2.**
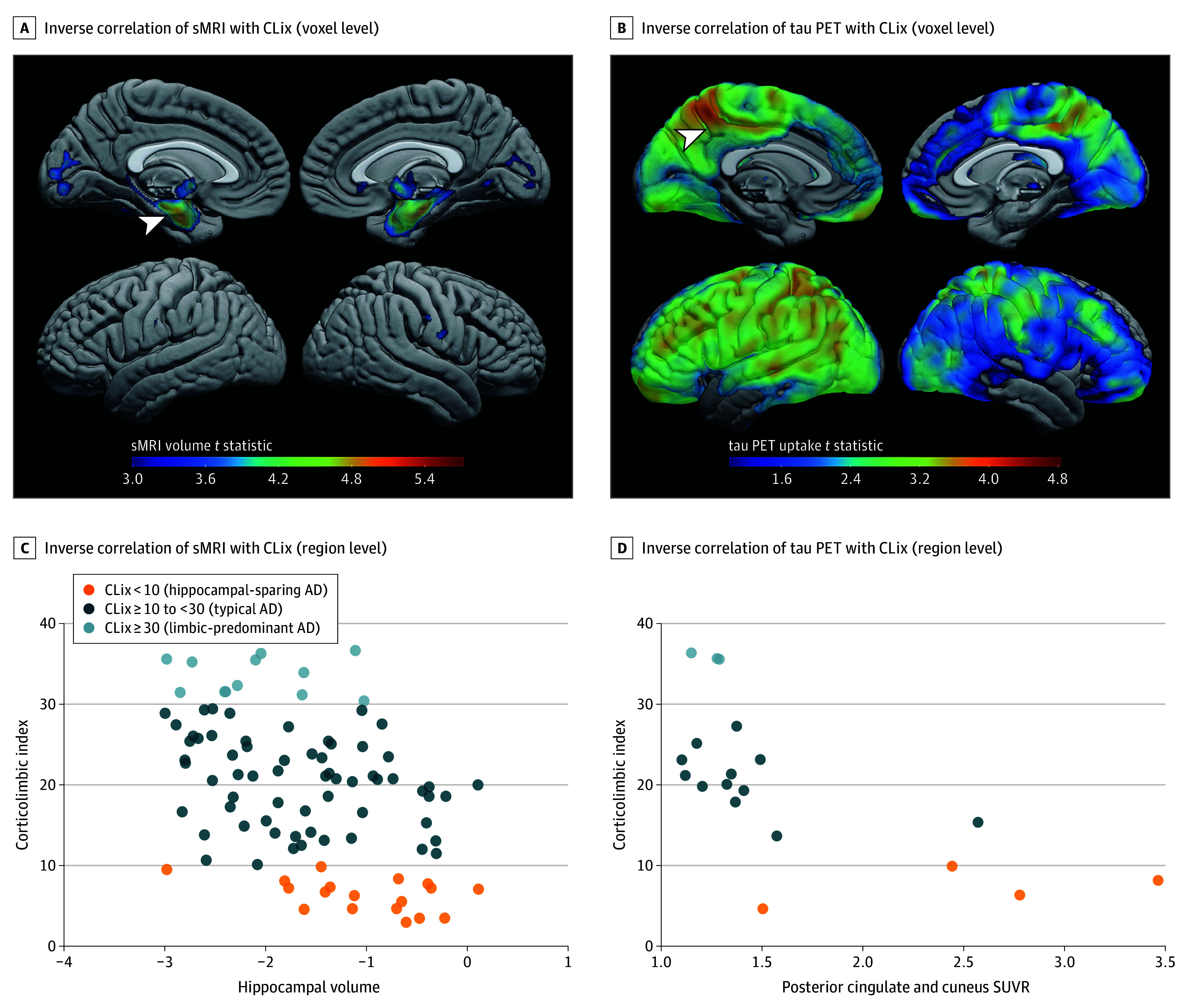
Association Between Structural Magnetic Resonance Imaging (sMRI) and Tau–Positron Emission Tomography (PET) With Corticolimbic Tangle Distribution A, Mapping the inverse association between structural volume from 3T MRI and corticolimbic index (CLix) demonstrates greater medial temporal lobe volume associating with lower CLix score consistent with a hippocampal sparing Alzheimer disease (AD) phenotype. The color bar indicates the value of the T statistic with greater volume loss shown in warmer colors. The arrowhead on structural MRI points to hippocampus. The adjoining scatterplot (C) of hippocampal volume reveals a strong association with CLix, which shows that a lower hippocampal volume associated with higher CLix score consistent with limbic predominant AD phenotype. B, Mapping the inverse association between flortaucipir tau-PET uptake and CLix demonstrates significant uptake in extra-temporal lobe cortical structures consistent with higher tau load in hippocampal sparing AD. The color bar indicates the value of the T statistic with higher tracer uptake shown in warmer colors. The arrowhead on tau-PET map points to posterior cingulate and cuneus region. The adjoining scatterplot (D) of posterior cingulate and cuneus tau-PET uptake reveals a strong association with CLix, which shows that lower cortical tau-PET uptake in posterior cingulate and cuneus associated with higher CLix score consistent with a limbic predominant AD phenotype. SUVR indicates standard uptake value ratio.

**Figure 3.**
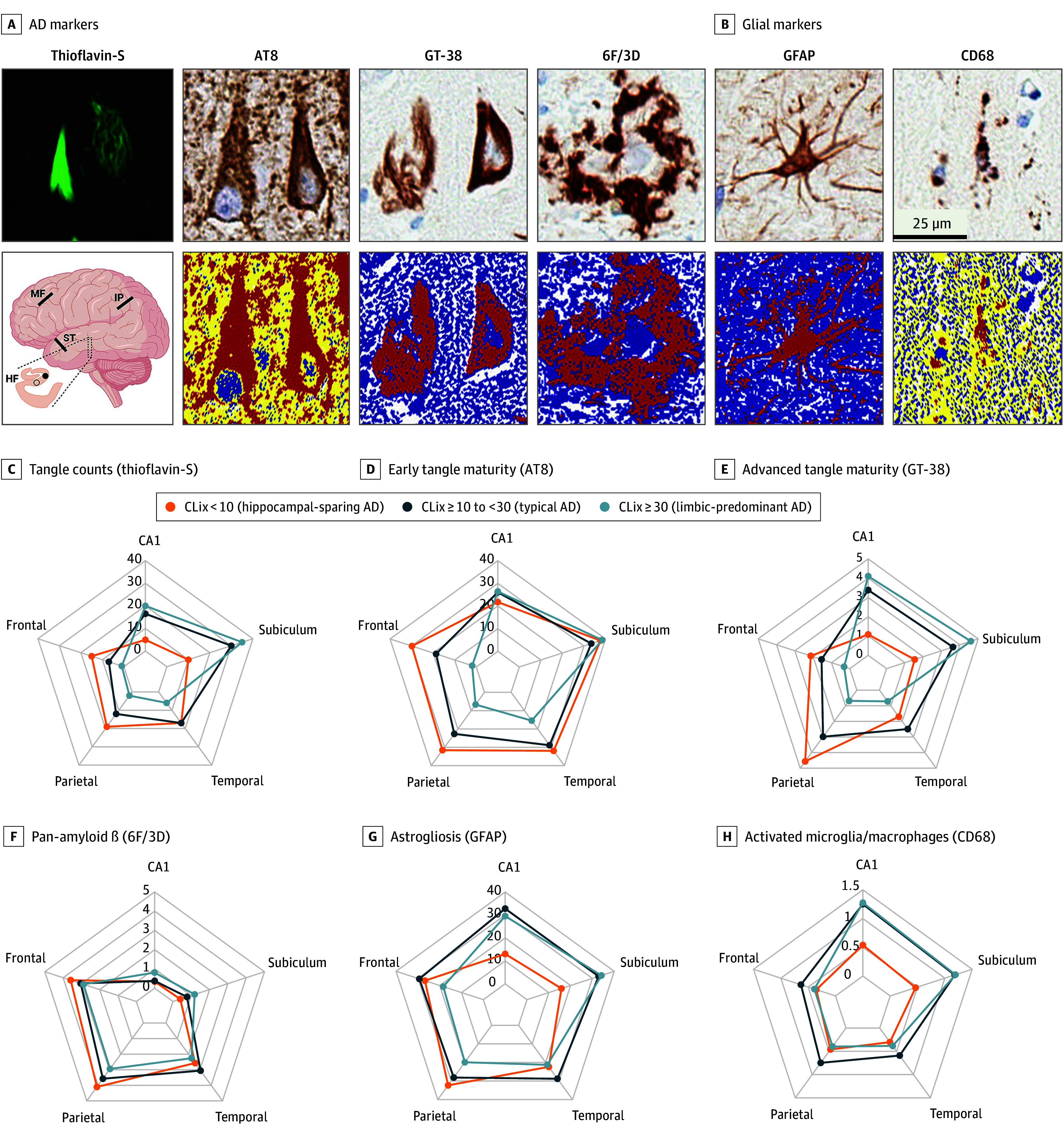
Digital Pathology Measures of Alzheimer Disease (AD) Pathology and Glial Activation Thioflavin-S fluorescent dye was used to manually count advanced neurofibrillary tangles, including mature tangles (left solid tangle) and ghost tangles (right tangle with splayed fibrils). Digital pathology was used to quantify markers of AD pathology and glial activation on serially stained 5-μm formalin-fixed, paraffin-embedded tissue sections in the digital pathology subgroup across 5 brain regions (note the illustration of the brain in panel A): CA1 (closed circle, hippocampus inset) and subiculum (open circle, hippocampus inset), as well as superior temporal, inferior parietal, and middle frontal association cortices. A, Thioflavin-S fluorescence microscopy was used to develop corticolimbic index (CLix) methodology and create the CLix R package (R Project for Statistical Computing) enabling quantification of AD corticolimbic tangle distribution. Antibodies used in this study included markers of hyperphosphorylated tau (phosphorylation-dependent anti-tau antibody 8 [AT8]), AD-specific tau conformers (anti-tau AD antibody [GT-38]), amyloid-β (6F/3D), astrogliosis (glial fibrillary acidic protein [GFAP]), and activated microglia/macrophages (CD68). A and B, The top row displays representative photomicrographs for each marker and the bottom row displays corresponding markup images of positive immunoreactivity (red on the markup images represents chromogen-positive pixels, blue represents negative pixels using the positive pixel count macro [GT-38, 6F/3D, and GFAP], and yellow and blue represent negative pixels for the color deconvolution macros [AT8 and CD68]). Radar plots are used to depict quantitative neuropathologic data with the axis increasing from the center (zero) to the circumference of the plot maxing at highest median. Higher scores signify higher number of thioflavin-S tangle counts or higher-percentage immunopositive staining. Scale bar for each panel = 25 μm. Brain image was created with BioRender.com. IP indicates inferior parietal; MF, middle frontal; ST, superior temporal.

In this cross-sectional study, the 3 study groups were formed by 2 neuropathologically diagnosed AD case series: the FLAME-AD series (Figure 1) and an independent neuroimaging group (Figure 2) who underwent antemortem 3T MRI and/or tau-PET (eFigure 1 in Supplement 1). A subgroup from FLAME-AD was used to derive the third study group for digital pathology analyses (Figure 3). All research was conducted on postmortem samples that are regarded by the Mayo Clinic institutional review board as exempt from the requirements of research on human participants. All brains were acquired with appropriate ethical approval, and the study was approved by the Mayo Clinic institutional review board. This study followed the Strengthening the Reporting of Observational Studies in Epidemiology (STROBE) reporting guidelines.

The FLAME cohort^13,22^ (n = 2809; 51% males; 49% females; age range, 36-104 years) housed at the Mayo Clinic brain bank in Jacksonville, Florida, was used to identify AD cases for the FLAME-AD series included in this study. Participating Memory Disorder Clinics in the State of Florida’s Alzheimer Disease Initiative provide the opportunity to register individuals for autopsy regardless of sex, ethnicity, or race. Studies deriving from the FLAME cohort aim for inclusivity by providing self-reported sex, ethnicity, and race. The race and ethnicity categories of the decedents were as follows: Asian, Black or African American, Hispanic or Latin American, Native American, and non-Hispanic White. The major requirement is that a documented neurologic or psychiatric workup for cognitive disorders be available. Referrals may also include educational talks to the community by Memory Disorder Center staff and family members of the brain bank participants. All individuals in this study have come to autopsy and are thus referred to as decedents. From the FLAME cohort, we excluded study brains that did not have AD as the primary neuropathologic diagnosis, were neuropathologically normal, or lacked thioflavin-S tangle data as these could not be subtyped. We further excluded AD cases with hippocampal sclerosis defined by disproportionate neuronal loss and gliosis in the hippocampus compared with observed tangles at the time of neuropathologic examination,^2,23^ as this coexisting neurodegenerative process interferes with subtype classification. Clinical diagnosis was not used as an inclusion/exclusion criterion to derive the final FLAME-AD series of decedents.

The FLAME-AD series was used to formulate an innovative approach to capturing spatially distributed thioflavin-S tangle counts. Reference percentiles from the posterior hippocampus (CA1, subiculum), association cortices (superior temporal, inferior parietal, middle frontal) (eTable 4 in Supplement 1), and the ratio of hippocampal to cortical tangle counts was used to account for the individuality of corticolimbic tangle distributions in AD. CLix is examined as a continuous trait that rescales percentiles of tangle distribution to a score ranging from 0 to 40 (eMethods 1 in Supplement 1). The CLix R package^24^ outputs a single score for each case that can be used to bin AD subtypes: less than 10 indicates relative cortical predominance/hippocampal sparing AD, 10 to 30 indicates typical AD, or 30 or greater indicates relative cortical sparing/ limbic predominant (eTable 4 in Supplement 1). Demographics and disease progression were retrospectively collected from clinical records provided to the brain bank by patients and/or next of kin (eMethods 2 in Supplement 1).

We then investigated an independent neuroimaging group derived from the Mayo Clinic Alzheimer Disease Research Center (ADRC) and Mayo Clinic Study of Aging (MCSA). Autopsied ADRC and MCSA study participants with antemortem neuroimaging performed within 3 years of death who had thioflavin-S tangle counts were analyzed (eMethods 5 in Supplement 1). The neuroimaging group included individuals with 3T MRI and another group with tau (flortaucipir)–PET, noting an overlap of cases between neuroimaging modalities.

FLAME-AD was used to select a subgroup of autopsied individuals for deep phenotyping with digital pathology to evaluate per AD subtype. Several exclusion criteria were applied to the FLAME-AD series to reduce cases to comprise the digital pathology subgroup. Copathologies that may contribute to glial activation, such as meningitis, encephalitis, (micro)infarction, or Lewy body disease, were excluded. CLix was used to select cases at the extreme ends of the corticolimbic continuum for hippocampal-sparing AD and limbic-predominant AD, with the centralized scores selected for typical AD.

### Procedures

Tissue samples were obtained during standardized neuropathologic evaluation as previously described^13^ and further detailed in eMethods 3 in Supplement 1. Thioflavin-S fluorescent dye was used to count tangles in all study groups. The antibodies, dilution factors, and pretreatments used in the digital pathology subgroup are listed in eTable 5 in Supplement 1. Immunohistochemistry was performed on serial tissue sections (eTable 6 in Supplement 1) and digitized in the digital pathology subgroup, on which annotations were drawn to facilitate quantification of neuropathologic burden (Figure 3 and eMethods 3 and eTable 6 in Supplement 1). Immunopositivity for transactive-response DNA-binding protein of 43 (TDP-43) was determined in the amygdala with TDP-43 distribution further assessed in the digital pathology subgroup using limbic predominant age-related TDP-43 encephalopathy neuropathologic change (LATE-NC staging).^25^ TaqMan single-nucleotide variant genotyping assays on DNA extracted from frozen tissue were used to determine *APOE* genotypes.^2^ NeuroChip (Illumina) using DNA extracted from frozen tissue was used to determine *TREM2* R47H variant status (eMethods 4 in Supplement 1).^26^ Analysis methods for 3T MRI processing and flortaucipir PET processing for standard uptake value ratio (SUVR) in the neuroimaging group are further elaborated on in eMethods 5 in Supplement 1.

### Statistical Analyses

Statistical analysis was performed using R statistical software, version 4.2.2 (R Foundation for Statistical Computing). Spearman rank correlation tested associations between CLix scores and clinicopathologic variables as continuous measures. Demographics and clinicopathologic characteristics among CLix-subtyped AD cases were tested using the Kruskal-Wallis rank sum test for continuous measures and the Fisher exact test for categorical measures. Post hoc comparisons between subtypes were performed with Wilcoxon rank sum testing. Partial Spearman correlations were reported for neuroimaging markers, which involved an adjustment of time from scan to death. All tests were 2-sided, and *P* values <.05 were regarded as statistically significant. To evaluate the importance of clinicopathologic heterogeneity measures, a random forest regression was established with an ensemble of 500 trees to form a forest of variable importance via the randomForest R package. The percentage increasing mean squared error (%IncMSE) was used to rank variable importance that was interpreted as the percentage increase in the MSE of the model if that variable was excluded. Study data were analyzed from December 2022 to December 2023.

## Results

The FLAME cohort included 2809 autopsied individuals; a total of 1448 were excluded (excluded study brains did not have AD as the primary neuropathologic diagnosis [n = 1084], were neuropathologically normal [n = 121], lacked thioflavin-S tangle data as these could not be subtyped [n = 101], carried a known AD gene variant [n = 18], or had hippocampal sclerosis [n = 124]) (eTable 1 in Supplement 1). Removal of exclusions resulted in 1361 neuropathologically diagnosed AD cases. A digital pathology subgroup of 60 FLAME-AD cases was derived for glial activation analyses (to evaluate 20 per AD subtype) (eTable 3 in Supplement 1). Antemortem neuroimaging was available in 93 Mayo Clinic study participants who came to autopsy with 3T MRI and 19 with tau (flortaucipir) PET, noting an overlap of 18 cases between neuroimaging modalities (eTable 2 in Supplement 1).

Among the 1361 FLAME-AD cases, 633 were male (47%; median [range] age at death, 81 [54-96] years), and 728 were female (53%; median [range] age at death, 81 [53-102] years). The race and ethnicity categories of included decedents were as follows: 1 Asian (0.1%), 14 Black or African American (1.0%), 62 Hispanic or Latin American (4.6%), 2 Native American (0.2%); and 1282 non-Hispanic White (94.2%).

### Clinicopathologic Heterogeneity in Neuropathologically Diagnosed AD Cases

To quantitatively investigate the association between measures of clinicopathologic heterogeneity and corticolimbic tangle distributions, CLix was evaluated as a continuous trait (Table). CLix was additionally used to bin subtypes to aid in graphical interpretation (Figure 1) and the reporting of case characteristics (eTable 1 in Supplement 1). The histogram plot for the FLAME-AD series visually displays the frequency of CLix score with hippocampal sparing AD (low CLix) and limbic-predominant AD (high CLix) shown as extreme corticolimbic phenotypes (Figure 1A). One Asian decedent had a CLix score of 22, Black or African American decedents had a median CLix score of 25, Hispanic/Latin American decedents had a median CLix score of 20, 2 Native American decedents had a CLix score of 18 and 22, and non-Hispanic White decedents had a median CLix score of 20 (eTable 7 in Supplement 1).

**Table.** Comparisons of Clinicopathologic Heterogeneity Measures and Variable Importance With Corticolimbic Tangle Distribution

Characteristics/continuous measures^a^	Clinicopathologic comparisons with CLix		Random forest regression, % increasing MSE^b^
	Spearman ρ	*P* value	
Antemortem findings			
Education, y	−0.11	.002	4.1
Age at symptomatic onset, y	0.39	<.001	46
Disease duration, y	0.07	.02	25
MMSE decline, points lost/y	0.27	<.001	NA^c^
Postmortem findings			
Age at death, y	0.43	<.001	NA^d^
Brain weight, g	−0.001	.98	8.5
Braak tangle stage	−0.18	<.001	24
Thal amyloid phase	0.01	.85	4.2
Kalaria CVD scale	0.10	<.001	3.6
Antemortem findings,^e^ median (IQR)			
Sex			
Male	18 (12-25)	<.001	9.2
Female	22 (16-28)		
APOE ε4 carriership			
APOE ε4−	18 (12-24)	<.001	8.4
APOE ε4+	21 (15-28)		
Atypical clinical syndrome^f^			
Atypical	13 (9-18)	<.001	14
Not	21 (15-27)		

Abbreviations: CLix, corticolimbic index; CVD, cerebrovascular disease; MMSE, Mini-Mental State Examination; MSE, mean squared error; NA, not available.

^a^ Spearman correlation coefficients were generated and *P* values resulted from the correlation test.

^b^ The percentage increasing MSE values were generated from a random forest regression model to uncover the importance of clinicopathologic variables contributing to a lower CLix score.

^c^ Due to missingness, MMSE decline was not included in the random forest regression model.

^d^ Due to collinearity with age at symptomatic onset, age at death was not included in the random forest model. Sensitivity test with age at death revealed similar level of importance (data not shown).

^e^ Median CLix scores (IQR) were generated and *P* values representing comparisons between characteristics resulted from the Wilcoxon rank-sum test.

^f^ An atypical clinical syndrome was classified for individuals with an antemortem clinical diagnosis of primary progressive aphasia, frontotemporal dementia, posterior cortical atrophy, corticobasal syndrome, or other less common diagnoses.

In the FLAME-AD series, a younger age at onset of cognitive complaints correlated with a lower CLix score (Spearman ρ = 0.39; *P* < .001) (Figure 1B), with young-onset AD (median [IQR] age, 16 [10-23] years) having a lower CLix score than late-onset AD (median [IQR] score, 22 [16-28]). A shorter disease duration (Spearman ρ = 0.07; *P* = .02) and higher education (Spearman ρ = −0.11; *P* = .002) correlated with a lower CLix score. An atypical, nonamnestic clinical syndrome was associated with a lower CLix score (median [IQR] score, 13 [9-18]) vs not atypical (median [IQR] score, 21 [15-27]; *P* < .001) (Figure 1C). Of note, a recently described dysexecutive syndrome in AD^27,28^ was retrospectively evaluated in the clinical records of the neuroimaging group given the level of detail provided by tertiary clinic specialists. Of the 15 AD cases presenting with dysexecutive syndrome, 8 (53%) had a CLix score less than 10, and all had a score less than 25 (eTable 2 in Supplement 1).

In the FLAME-AD series, males had a lower CLix score (median [IQR] score, 18 [12-25]) than females (median [IQR] score, 22 [16-28]; *P* < .001). A more rapid rate of cognitive decline also correlated with a lower CLix score (Spearman ρ = 0.27; *P* < .001). *APOE* ε4 noncarriers (median [IQR] score, 18 [12-24]) had a lower CLix score than *APOE* ε4 carriers (median [IQR] score, 21 [15-28]; *P* < .001), whereas *TREM2* R47H carriers (31 of 972 [3.2%]; median [IQR] score, 16 [11-22]) had a lower CLix score than noncarriers (941 of 972 [96.8%]; median [IQR] score, 20 [14-27]; *P* = .02). Cases with a nonamnestic syndrome had lower CLix scores (median [IQR] score, 13 [9-18]) vs not (median [IQR] score, 21 [15-27]; *P* < .001). A younger age at death correlated with a lower CLix score (Spearman ρ = 0.43; *P* < .001). A higher Braak stage (Spearman ρ = −0.18; *P* < .001) and lower Kalaria cerebrovascular disease scale score (Spearman ρ = 0.10; *P* < .001) were correlated with a lower CLix score. Neither brain weight nor Thal phase correlated with CLix score. TDP-43 negative cases (median [IQR] score, 16 [10-23]) were associated with a lower CLix score than TDP-43 positive cases (median [IQR] score, 20 [16-28]; *P* < .001).

### Importance of Clinicopathologic Heterogeneity Measures to Corticolimbic Tangle Vulnerability

A random forest regression model was used to investigate the variable importance of these clinicopathologic heterogeneity measures to corticolimbic tangle vulnerability as a continuous trait (Table). Age at symptomatic onset was the most important predictor, with a 46% increase in MSE of model if excluded as a factor (%IncMSE = 46.2). Exclusion of disease duration (%IncMSE = 25.4) and Braak stage (%IncMSE = 24.1) from the model would result in a 25% and 24% increase in error of the model, respectively. The next highly ranked importance variable was an atypical clinical syndrome that would result in 14% increase in error of the model (%IncMSE = 14.5). The remaining factors fell below 10% increase in error of the model if excluded: male sex (%IncMSE = 9.24), *APOE ε4* carriership (%IncMSE = 8.45), brain weight (%IncMSE = 8.50), Thal amyloid phase (%IncMSE = 4.17), and Kalaria cerebrovascular disease scale (%IncMSE = 3.61).

### Neuroimaging Correlates With Corticolimbic Tangle Vulnerability

In an independent neuroimaging group of 93 study participants (n = 93 MRI, n = 19 tau-PET]) (eTable 2 in Supplement 1), greater hippocampal 3T MRI volume adjusted by MRI date to death correlated with lower CLix score (Spearman ρ = −0.45; *P* < .001) (Figure 2A and eFigure 2 in Supplement 1). Higher cortical flortaucipir PET SUVR adjusted by PET date to death was also found to correlate with lower CLix score (21 of 93 [22.6%]) (Figure 2B and eFigure 2 in Supplement 1), as exampled by parietal cortex (Spearman ρ = −0.72; *P* < .001) and posterior cingulate and precuneus cortex (Spearman ρ = −0.74; *P* < .001).

### Regional Glial Activation Patterns Among Corticolimbic Subtypes of AD

A digital pathology subgroup (n = 60) from FLAME-AD was selected to more deeply phenotype glial activation patterns using CLix score to subtype AD for group comparisons (Figure 3 and eFigure 3 in Supplement 1). Frontoparietal and hippocampal patterns of tau and amyloid-β immunohistochemical burden are briefly described to contextualize findings with regional data provided in eTable 8 in Supplement 1. Thioflavin-S tangle counts and percentage of GT-38 AD-tau conformer burden revealed similar monotonically directed corticolimbic distribution resembling intersecting pentagons. Analysis of percentage of AT8 hyperphosphorylated tau burden did not uncover hippocampal differences, but distinct cortical distribution was found. Although corticolimbic amyloid-β distribution was uniformly stereotyped with a tight formation of overlapping pentagons, hippocampal differences were found.

To provide a more in-depth evaluation of glial activation patterns, the CA1 hippocampal subsector and inferior parietal cortex data will be described with post hoc *P* values. The percentage of GFAP astrogliosis burden was lowest in the hippocampus of hippocampal sparing AD (median [IQR], 13% [8.5%-18%]) but plateaued in typical AD (median [IQR],33% [22%-43%]; post hoc *P* < .001) and limbic predominant AD (median [IQR], 30% [28%-40%]; post hoc *P* < .001) relative to monotonic increase in percentage of GT-38 burden. The cortical percentage of GFAP burden was lowest in limbic predominant AD (median [IQR], 20% [18%-24%]) but plateaued in typical AD (median [IQR], 28% [24%-33%]; post hoc *P* < .001) and hippocampal sparing AD (median [IQR], 32% [23%-40%]; post hoc *P* < .001). The hippocampal percentage of CD68 activated microglia/macrophages burden was lowest in hippocampal sparing AD (median [IQR], 0.54% [0.39%-0.79%]) but plateaued in typical AD (median [IQR], 1.2% [0.96%-1.8%; post hoc *P* < .001) and limbic predominant AD (median [IQR], 1.3% [0.94%-1.5%]; post hoc *P* < .001). The cortical percentage of CD68 burden was lower in limbic predominant AD (median [IQR], 0.40% [0.32%-0.57%]) compared with typical AD (median [IQR], 0.75% [0.51%-0.98%]; post hoc *P* < .004). The cortical area with the highest tangle count and tau burden in hippocampal sparing AD was not found to differ for percentage of CD68 burden (median [IQR], 0.46% [0.32%-0.75%]) compared with either typical AD (median [IQR], 0.75% [0.51%-0.98%]; post hoc *P* = .06) or limbic predominant AD (median [IQR], 0.40% [0.32%-0.57%]; post hoc *P* = .37).

## Discussion

In this cross-sectional study of neuropathologically diagnosed AD, we sought to examine the importance of clinicopathologic heterogeneity and evaluate glial activation patterns along a continuum of corticolimbic tangle distribution. CLix score was associated with relevant demographic and clinicopathologic observations in more than 1300 autopsied FLAME-AD cases. CLix score was further validated in a prospectively followed Mayo Clinic neuroimaging group, where a lower CLix score associated with greater medial temporal lobe volume and higher cortical tau-PET uptake. The utility of the CLix for deep phenotyping was demonstrated using digital pathology in 60 AD cases that revealed distinct brain region and cell type-specific differences in glial activation among AD subtypes. Compared with typical AD and limbic predominant AD, AD cases with relative hippocampal sparing had lower CD68 burden in association cortices, which suggests a reduction in activated microglia/macrophages. Reduced activated microglia/macrophages in the cortex of hippocampal sparing AD was observed despite having the highest cortical tau burden.

The most important antemortem and postmortem factor predicting corticolimbic tangle distribution was age at symptomatic onset and age at death. A lower CLix score was more common in young-onset AD who present with cognitive impairment before the age of 65 years and lack a known autosomal dominant gene variant. The utility of the CLix as a continuous trait was also demonstrated in AD cases presenting with an atypical clinical syndrome in which disproportionate cortical tangle pathology (ie, lower CLix score) was found in individuals with an affected behavioral, executive, praxis, language, or visuospatial domain.^2,5^ As cortical tau accumulation increases, these patients are progressively unable to perform activities of daily living and are found to decline at a faster rate than similarly aged patients with amnestic AD.^5^ Future studies will focus on better capturing the recently described dysexecutive syndrome in AD^27^; as retrospective examination in the neuroimaging group revealed a large increase in the frequency of atypical clinical syndromes in hippocampal sparing AD from 50% to 89% when dysexecutive AD was considered. Patients with young-onset AD are more commonly observed to have an atypical, nonamnestic clinical syndrome where cortical tau pathology and antemortem tau-PET is observed to be higher.^5,29^ Taken together, our data extend our previous work that now demonstrates corticolimbic tangle distributions as a flattened score reflecting a constellation of clinically meaningful information that may aid interpretation of medical history.

GFAP burden in AD brains was found to plateau in the hippocampal subsectors of limbic predominant AD and association cortices of hippocampal sparing AD relative to areas of highest burden of GT-38 AD-tau conformer. Astrogliosis, immunohistochemically measured by GFAP, was previously reported to be higher in areas of amyloid-β and tau pathology.^30^ Astrocytic processes penetrate extracellular ghost tangles, resulting in an eosinophilic appearance on routine hematoxylin-eosin–stained sections.^31^ However, we speculate that the observed plateauing occurring in high-density areas of ghost tangles may reflect a reduction in astrocyte hypertrophy and astrocyte activation owing to the lack of an injury signal coming from dead tangle-bearing neurons.

Evaluation of activated microglia/macrophages in the hippocampus also found a plateauing of CD68 burden in limbic predominant AD brains that could be suggestive of a saturation point for microglial reactivity to tau-mediated neurodegeneration. Further inspection of cortical patterns of CD68 in areas of the highest burden of AD-tau conformers measured by GT-38 revealed a blunting in the association cortices of hippocampal sparing AD. Our findings suggest lower cortical levels of activated microglia/macrophages, offering fewer protective functions of microglia,^32^ may contribute to the distinct clinical course in hippocampal sparing AD patients. Regional variability in cortical tau and amyloid-β burden,^4,7^ along with activated microglia/macrophages remains a critical area of study especially in the context of atypical, nonamnestic AD clinical presentations.^33,34,35^ Our findings suggest that AD brains with lower CLix score may reveal a distinct activated microglia/macrophages signature specific to the hippocampal sparing AD phenotype, which motivates future studies to consider relevance of syndromic presentation.

We report an innovative genotype-phenotype association analysis between *TREM2* R47H variant carriers and lower CLix score. Our finding extends previous reports of a greater frequency of *TREM2* variants in AD cases who presented clinically with atypical, nonamnestic syndromes.^36,37^ Activated microglia/macrophages is a key process of the innate immune response that is modulated by *TREM2*, a protein-coding gene highly expressed in microglia.^14,38^ It will be of further interest to consider additional genetic contributions to microglial/macrophage deficiency that we hypothesize as underlying selective cortical vulnerability to tangle pathology in atypical AD, especially in hippocampal sparing AD cases (ie, low CLix score), who have a lower frequency of *APOE ε4* carriership.^3^ Future genetic studies investigating corticolimbic vulnerability AD will seek to substratify by key demographic phenotypes (eg, young onset vs late onset) and cortical predominance (eg, parietal vs temporal) to identify if within subtype variability in microglial/macrophage deficiency associates with genetic variability.

Through our investigation of antemortem neuroimaging from participants with neuropathologically defined CLix scores, we found that greater hippocampal atrophy on structural MRI was associated with higher CLix score and greater cortical tau-PET uptake was associated with lower CLix score. These results support the potential clinical utility of CLix scores in guiding the development of biomarkers for classification of AD subtypes. In vivo evidence of AD subtypes is supported by recent tau-PET studies that used flortaucipir to investigate tau distribution.^39,40^ The current study provides a backward engineering of foundational knowledge gained by examining the postmortem brain to be applied to antemortem neuroimaging modalities. We hypothesize that the translation of thioflavin-S corticolimbic distribution to flortaucipir tau-PET remained robust as the radioligand recognizes advanced tangle maturity.^41,42^

### Strengths and Limitations

The main strength of the current study is the use of human brain tissue in conjunction with antemortem and postmortem measures of heterogeneity. Our findings extend our original analysis of heterogeneity in AD^2^ in a larger sample size that now includes a quantitative measure of corticolimbic vulnerability as a continuous trait, innovative genotype-phenotype associations with *APOE* and *TREM2*, application of random forest regression to identify importance of clinicopathologic factors of heterogeneity, neuroimaging maps in an independent series, digital pathology analysis of glial activation patterns. This enabled us to concretely study neuropathologically diagnosed AD brains but may limit extrapolation to early disease course. Although we do capture posterior cortical involvement by including the inferior parietal cortex into the CLix calculation, addition of the occipital cortex into the equation may prove to be useful to fully characterize AD cases with posterior cortical atrophy. GFAP and CD68 were chosen as robust immunohistochemical markers in human brains to study glial activation patterns, which have the potential to inform plasma biomarker studies. However, evaluation of GFAP and CD68 may only represent a subpopulation of disease-associated glial activation. Although a useful antibody for recognizing AD-tau conformers, we found that the GT-38 antibody recognition precipitously dropped off in areas of end-stage ghost tangles. Validation of CLix through visualization on MRI and tau-PET provides supportive evidence of future translation to the clinic, although more work will be needed to assess in the context of disease severity.

## Conclusions

In summary, results of this cross-sectional study suggest that clinicopathologic heterogeneity and glial activation patterns were associated with corticolimbic tangle distribution. CLix score was useful in binning AD subtypes but also enabled the enrichment of extreme and representative corticolimbic phenotypes. Extension of the CLix score using neuroimaging modalities (eg, MRI, tau-PET) will require consideration of disease stage and severity as the current study took place in the context of advanced AD. Our findings also have important implications for the design and interpretation of clinical trials, as recognition of relational corticolimbic tangle distributions may inform clinical readouts of cognition or biomarker changes. Moreover, the observed microglial/macrophage deficiency in hippocampal sparing AD highlights the need for personalized combination therapies that target chronic immune dysregulation.

## Notes

Supplement 1. **eMethods 1.** Corticolimbic Index (CLix) Development **eMethods 2.** Retrospective Clinical Abstraction **eMethods 3.** Neuropathologic Procedures and Digital Pathology **eMethods 4.** TREM2 R47H Variant **eMethods 5.** ADRC and MCSA Study Cohort Descriptions, Clinical and neuroimaging Procedures, and Neuropathologic Procedures **eTable 1.** Clinicopathologic Characteristics of the FLAME-AD Series Used to Develop the CLix Methodology for AD **eTable 2.** Clinicopathologic Characteristics of the Neuroimaging Group **eTable 3.** Clinicopathologic Characteristics of the Digital Pathology Subgroup **eTable 4.** Reference Percentiles and Thioflavin-S Positive Tangle Counts for AD Subtype Classifier Algorithm **eTable 5.** Characteristics of Primary Antibodies Used in this Study **eTable 6.** Slides Stained and Evaluated for the Digital Pathology Subgroup **eTable 7.** CLix Distribution Among AD Subtypes Stratified by Ethnoracial Status **eTable 8.** Regional Neuropathologic Measures in the AD Digital Pathology Subgroup **eFigure 1.** Overview of 3 Study Groups Evaluated With CLix **eFigure 2.** Structural MRI and Tau-PET Scans Across CLix Scores **eFigure 3.** Regional Quantitative Measures and Distributions in the Digital Pathology Subgroup Among AD Neuropathologic Subtypes
eReferences.

Supplement 2.
Data Sharing Statement.
