# Supplementary material for: Clinicopathologic Heterogeneity and Glial Activation Patterns in Alzheimer Disease
Source: JAMA Neurol. 2024 Apr 15;81(6):619–29. doi: 10.1001/jamaneurol.2024.0784 (PMC11019448; doi:10.1001/jamaneurol.2024.0784)
Supplement: Supplement 1. — eMethods 1. Corticolimbic Index (CLix) Development eMethods 2. Retrospective Clinical Abstraction eMethods 3. Neuropathologic Procedures and Digital Pathology eMethods 4. TREM2 R47H Variant eMethods 5. ADRC and MCSA Study Cohort Descriptions, Clinical and neuroimaging Procedures, and Neuropathologic Procedures eTable 1. Clinicopathologic Characteristics of the FLAME-AD Series Used to Develop the CLix Methodology for AD eTable 2. Clinicopathologic Characteristics of the Neuroimaging Group eTable 3. Clinicopathologic Characteristics of the Digital Pathology Subgroup eTable 4. Reference Percentiles and Thioflavin-S Positive Tangle Counts for AD Subtype Classifier Algorithm eTable 5. Characteristics of Primary Antibodies Used in this Study eTable 6. Slides Stained and Evaluated for the Digital Pathology Subgroup eTable 7. CLix Distribution Among AD Subtypes Stratified by Ethnoracial Status eTable 8. Regional Neuropathologic Measures in the AD Digital Pathology Subgroup eFigure 1. Overview of 3 Study Groups Evaluated With CLix eFigure 2. Structural MRI and Tau-PET Scans Across CLix Scores eFigure 3. Regional Quantitative Measures and Distributions in the Digital Pathology Subgroup Among AD Neuropathologic Subtypes eReferences. [file jamaneurol-e240784-s001.pdf]

Supplementary Online Content

Kouri N, Frankenhauser I, Peng Z, et al. Clinicopathologic heterogeneity and glial activation patterns in Alzheimer disease. *JAMA Neurol*. Published online April 15, 2024. doi:10.1001/jamaneurol.2024.0784

eMethods 1. Corticolimbic Index (CLix) Development

eMethods 2. Retrospective Clinical Abstraction

eMethods 3. Neuropathologic Procedures and Digital Pathology

eMethods 4. TREM2 R47H Variant

eMethods 5. ADRC and MCSA Study Cohort Descriptions, Clinical and neuroimaging Procedures, and Neuropathologic Procedures

eTable 1. Clinicopathologic Characteristics of the FLAME-AD Series Used to Develop the CLix Methodology for AD

eTable 2. Clinicopathologic Characteristics of the Neuroimaging Group

eTable 3. Clinicopathologic Characteristics of the Digital Pathology Subgroup

eTable 4. Reference Percentiles and Thioflavin-S Positive Tangle Counts for AD Subtype Classifier Algorithm

eTable 5. Characteristics of Primary Antibodies Used in this Study

eTable 6. Slides Stained and Evaluated for the Digital Pathology Subgroup

eTable 7. CLix Distribution Among AD Subtypes Stratified by Ethnoracial Status

eTable 8. Regional Neuropathologic Measures in the AD Digital Pathology Subgroup

eFigure 1. Overview of 3 Study Groups Evaluated With CLix

eFigure 2. Structural MRI and Tau-PET Scans Across CLix Scores

eFigure 3. Regional Quantitative Measures and Distributions in the Digital Pathology Subgroup Among AD Neuropathologic Subtypes

eReferences.

This supplementary material has been provided by the authors to give readers additional information about their work.

© 2024 Kouri N et al. *JAMA Neurology*.

eMethods

eMethods 1. CorticoLimbic index (CLix) development

We have previously defined a mathematical algorithm to classify Alzheimer's disease (AD) subtypes in our discovery cohort of 889 AD cases based on their corticolimbic patterns of tangle accumulation.[3] However, a conceptual method was lacking that would enable us to visualize how extreme a hippocampal sparing AD or limbic predominant AD case was relative to a more representative typical AD case. By removing the extreme AD phenotypes, we would theoretically be better positioned to reduce attributable variance and improve our ability to interpret relevant clinicopathologic findings in the more common form of typical AD. To account for the individuality of corticolimbic patterns in AD, we introduce a novel re-expression of the AD subtype algorithm as a continuous index termed CLix.

As previously described in the discovery cohort,[3] tangle counts were used for subtyping AD cases based on corticolimbic patterns of tangle accumulation. The posterior hippocampus at the level of the lateral geniculate nucleus was reviewed to obtain tangle counts in the CA1 and subiculum. Cortical tangle counts were obtained from association cortices, including superior temporal, inferior parietal, and middle frontal. The original algorithm assessed the regional severity of tangle accumulation in an individual compared to that in the overall AD cohort, as well as the ratio of hippocampal subsector accumulation to cortical regions within each case. In the refined algorithm, we used data from AD cases in the larger Florida Autopsied Multi-Ethnic AD (FLAME-AD) series (n=1361) as the new reference population and re-expressed the algorithm in terms of a continuous numeric score that we refer to as CLix.

The original discovery cohort overlaps with the FLAME-AD series by 54% (n=739/1361). The algorithm refers to percentiles of tangle count distributions and derived quantities, noting that the 50th percentile is the median (eTable 4). Tangle counts in the hippocampal subsectors (H) and cortical regions (C) were used, both being integral to the implementation of Braak tangle staging.[5] The inputs of the algorithm are the hippocampal and cortical counts and their means, as well as the ratio (R) of the hippocampal and cortical means, all relative to their respective medians in the reference population. CLix re-expresses the algorithm as a continuous numeric value between 0 and 40 such that those with values of less than 10 are classified as hippocampal sparing AD, while those with values of 30 or higher are classified as limbic predominant AD (Figure 1A). The continuous measure is useful in that it places AD cases on a spectrum, with some for which the subtype is clear (<10: hippocampal sparing, ≥10 to <30: typical, ≥30: limbic predominant) while those with scores close to the cut-off of 10 or 30 are more borderline. Thus, even cases classified as typical AD can be assessed on a continuum.

The CLix creation algorithm and imputation for missing tangle counts values were described in detail within attached files on GitHub (Translational Neuropathology Lab. GitHub). To describe the CLix algorithm briefly. For a new case, we have $\boldsymbol{H_1^*}$ and $\boldsymbol{H_2^*}$ to represent the neurofibrillary tangle (NFT) counts in the hippocampal subsectors of CA1 and Subiculum; $\boldsymbol{C_1^*}$, $\boldsymbol{C_2^*}$ and $\boldsymbol{C_3^*}$ to represent the NFT counts in the cortical regions of superior temporal, inferior parietal and middle frontal.

$$\text{Hippocampal Mean: } H_3^* = \frac{H_1^* + H_2^*}{2}$$
$$\text{Cortical Mean: } C_4^* = \frac{C_1^* + C_2^* + C_3^*}{3}$$
$$\text{Ratio of Hippocampal to Cortical Means: } R^* = \frac{H_3^*}{C_4^*}$$

To assign a CLix score, this R^* is then measured in terms of the distribution of R from the reference dataset resulting in kR. kR is the proportion of the reference dataset that is less than R^*. We handle kR one of the following three ways:

- If kR is equal to 50, we multiply by 0.4 to rescale and account for kR being a proportion and the CLix score is calculated as 0.4 * kR.
- If kR is less than 50, the CLix score will be 0.4 times the maximum of kR, kH_L, and kC_L. kH_L is the largest of the proportion of H_i in the reference dataset that is less than H_i^* divided by 2, with $i = 1,2,3$. kC_L is the second largest of the proportion of C_j in the reference dataset that is greater than or equal to C_j^* divided by 2, with $j = 1,2,3,4$.
- If kR is greater than 50, the CLix score will be 0.4 times the minimum of kR, kH_U, and kC_U. kH_U is the second smallest of the proportion of H_i in the reference dataset that is greater than or equal to H_i^* divided by 2 and subtracted from 100, with $i = 1,2,3$. kC_U is the second smallest of the proportion of C_j in the reference dataset that is less than or equal to C_j^* divided by 2 and subtracted from 100, with $j = 1,2,3,4$.

eMethods 2. Retrospective clinical abstraction

We retrospectively collected data on demographics and clinical progression from existing clinical records provided to the brain bank by participants and/or family members. Years of education were collected. The age at onset of first cognitive symptoms was calculated by subtracting the date of birth from the approximate date at onset of cognitive symptoms. The date at onset was then subtracted from the

© 2024 Kouri N et al. *JAMA Neurology*.

date at death to calculate disease duration in years. Disease duration was calculated by subtracting the date at onset from the date of death and dividing the values by 365.25. An atypical clinical syndrome[6] was classified for individuals with an antemortem clinical diagnosis of primary progressive aphasia, frontotemporal dementia, posterior cortical atrophy, corticobasal syndrome, and other less common diagnoses. To assess cognitive impairment, we recorded both the date and score from Mini-Mental Status Examination (MMSE) testing. We specifically examined differences in the final MMSE, which was required to have been administered within three years of the date of death. Longitudinal decline on the MMSE was calculated as points lost per year in individuals with three or more MMSE test scores available.

eMethods 3. Neuropathologic procedures and digital pathology

Tissue samples were obtained during standardized neuropathologic evaluation of the brains by a single neuropathologist, Dennis W. Dickson, the director of the Mayo Clinic brain bank. Formalin-fixed paraffin-embedded tissue sections from the posterior hippocampus, superior temporal cortex, inferior parietal cortex, and middle frontal cortex were cut into 5 μm thin slices and mounted on positively charged glass slides (Figure 1). As previously described,[1] standard neuropathologic examinations and procedures were performed to determine the Braak tangle stage[5] and Thal amyloid phase.[7] Thioflavin-S, a fluorescent dye that binds to β-pleated sheets of which both tangles and amyloid-β plaques are composed, was used to assess tangle counts with a 40X objective (0.125 mm^2 microscopic field) and amyloid plaque density (to a maximum of 50 per microscopic field) with a 10X objective (3 mm^2 microscopic field) using an Olympus BH2 fluorescent microscope (Evident Corporation, Tokyo, Japan). Available data for TDP-43 positivity are reported for FLAME-AD from screening efforts performed in the amygdala. For the digital pathology subgroup, limbic predominant age-related TDP-43 encephalopathy neuropathologic changes (LATE-NC) staging was assigned to each case using TDP-43 immunohistochemistry on the amygdala, hippocampus, and middle frontal cortex.[8] Cerebrovascular disease was measured using the modified Kalaria scale,[9] as previously described.[10] We excluded cases in the digital pathology subgroup (n=60) with high cerebrovascular burden (ie, microinfarcts and/or infarcts) as indicated by a cortical score of 5 to 6 and/or basal ganglia score of 3 to 4.

The antibodies, dilution factors, and pretreatments employed in this study are listed in eTable 5 . Serial tissue slides (eTable 6) were stained using a Lab Vision Autostainer 480S (Thermo Scientific, Waltham, MA, USA) to detect hyperphosphorylated tau (AT8 [1:2500]; cat# MN1020, Thermo Scientific), AD-specific tau conformers (GT-38[11] [1:10000]; cat# ab246808; Abcam USA, Boston, MA, USA), amyloid-β (6F/3D [1:100]; cat# M0872, Agilent Technologies, Santa Clara, CA, USA), astrogliosis (GFAP [1:5000]; cat# MU020-UC, clone GA-5, BioGenex, Fremont, CA, USA), and activated microglia/macrophages (CD68 [1:750]; cat# M081401-2, clone KP1, Agilent Technologies). Amygdala sections were screened for TDP-43 pathology using the MC2085 antibody C-terminally cleaved TDP-43 ([1:3000] a gift from Leonard Petrucelli).[12] When performing LATE-NC staging in the digital pathology subgroup, hippocampal and frontal areas missing TDP-43 information were supplemented with immunostaining using phosphorylated TDP-43 (pTDP-43 at S409-410 [1:5000]; cat# CAC-TIP-PTD-M01A, clone 11-9, Cosmo Bio USA, Carlsbad, CA, USA) (eTable 5). 3,3-diaminobenzidine was used as the chromogen. Immunohistochemically stained slides were counterstained with hematoxylin and coverslipped using a Leica CV5030 Robotic Coverslipper (Leica Biosystems, Wetzlar, Germany).

Tissue slides from the posterior hippocampus, superior temporal, inferior parietal, and middle frontal cortices were scanned on an Aperio AT2 scanner (Leica Biosystems, Buffalo Grove, IL, USA) and ImageScope software (Leica Biosystems, version 12.4.2.7000) was used for drawing annotations to digitally quantify neuropathology. The hippocampal CA1 was traced using the lacunosum layer as the superior border, the alveus served as the inferior border, and the midway of the dentate gyrus separated CA1 from the subiculum. All stains of the same tissue sample were traced simultaneously with neuroanatomic guidance from diagnostic hematoxylin and eosin (H&E)-stained sections to ensure analysis of matching brain regions and pathologies. Sections of association cortices (superior temporal, inferior parietal, and middle frontal) were annotated in the gray matter using the strait of the gyrus, where the superficial surface and gray/white junction lay parallel, as a landmark for consistency across samples. All tracing of annotation layers was performed blinded to AD subtype. Two custom designed color-deconvolution macros (AT8 and CD68) and three positive-pixel count macros (GT-38, 6F/3D, GFAP) (Figure 1, eTable 5) were applied.[13] The macros identify positive staining for tau, amyloid-β, GFAP, and CD68 burden on tissue sections, which is then converted into a percent burden of the total area annotated that represents positive staining per area annotated. Representative images are provided in (Figure 1) displaying characteristic staining of the neuropathologic lesions and their corresponding macro markup images.

eMethods 4. *TREM2* R47H variant

Total DNA was extracted from frozen cerebellum using standard protocols with AutoGen FlexSTAR (AutoGen, Holliston, MA, USA). Cases were previously genotyped using NeuroChip (Infinium NeuroConsortium Array, Illumina, Inc., San Diego, CA, USA) to determine *TREM2* R47H variant status. Genotyping of the NeuroChip signal-intensity data was performed using GenomeStudio version 2.0.5 with default settings. Genotype calls were exported from GenomeStudio to PLINK[14] format and annotated using ANNOVAR.[14,15] Genotypes at the *TREM2* R47H position were then extracted.

© 2024 Kouri N et al. *JAMA Neurology*.

eMethods 5. ADRC and MCSA study cohort descriptions

Clinical and neuroimaging procedures

The neuroimaging group (n=93) was derived from Mayo Clinic Alzheimer's disease research center (ADRC) and Mayo Clinic Study of Aging (MCSA). The ADRC study participants are recruited through dementia referral services. Whereas the MCSA is a population-based, epidemiological cohort that was designed to investigate the prevalence, incidence, and risk factors for mild cognitive aging and dementia among the residents of Olmsted County, Minnesota[16]. Using the Rochester Epidemiology Project (REP) medical records linkage system infrastructure[17,18] to enumerate Olmsted County population, study participants were randomly invited to participate in the MCSA using an age- and sex-stratified sampling frame. Demographics and clinical progression measures were databased prospectively. To compare cognitive impairment findings to FLAME-AD, we examined longitudinal MMSE decline as points lost per year in individuals with three or more MMSE test scores available. The clinical diagnosis of the participants was ascertained at the time of magnetic resonance imaging (MRI) using previously published criteria.[19] An atypical clinical syndrome was classified for individuals with an antemortem clinical diagnosis of primary progressive aphasia, frontotemporal dementia, posterior cortical atrophy, and corticobasal syndrome. A recently described dysexecutive syndrome in AD[20] was retrospectively evaluated in the clinical records of the neuroimaging group.

All MRIs were acquired on 3T Siemens and GE scanners. The acquisition protocols and analysis for MRI (n=93) and tau positron emission tomography (PET, n=19/93) were previously published.[21,22] The voxel level analyses were conducted using unified segmentation in SPM12[23] with MCALT templates and settings for structural MRI and tau PET scans (https://www.nitrc.org/projects/mcalt/). For neuroimaging analyses, a false discovery rate was corrected at $p<0.05$ for MRI and for tau-PET. The significant voxels with the following thresholds were displayed using MRIcroGL software.

Neuropathologic procedures

Neuropathologic sampling followed Consortium to Establish a Registry for Alzheimer's Disease (CERAD) recommendations and National Institute on Aging-Alzheimer's Association criteria for AD neuropathologic change assessment.[24,25] Formalin-fixed, paraffin-embedded 5-µm-thick tissue sections were stained with hematoxylin and eosin, as well as Bielschowsky silver stain. As described in the eMethods, thioflavin-S microscopy was used to quantify the number of tangles in posterior hippocampal subsectors (CA1, subiculum), superior temporal, inferior parietal, and middle frontal. Tangles counts were inputted into the CLix package to assign a CLix score and AD subtype.

© 2024 Kouri N et al. *JAMA Neurology*.

eTables

eTable 1. Clinicopathologic characteristics of the FLAME-AD series used to develop the CLix methodology for AD

Characteristic	Alzheimer's disease neuropathologic subtypes (n=1361)			*P* value
	**Hippocampal sparing** (n=175, CLix <10)	**Typical** (n=1014, CLix ≥10 to <30)	**Limbic predominant** (n=172, CLix ≥30)	
Female, %	62/175 (35%)	545/1014 (54%)	121/172 (70%)	.001
Education, yr	16 (12,16)	14 (12,16)	14 (12,16)	.007
APOE ε4 carriership, %	68/147 (46%)	496/779 (64%)	105/144 (73%)	.001
**Clinical findings**				
Age onset of cognitive symptoms, yr	65 (56,72)	71 (65,77)	78 (72,81)	.001
Disease duration, yr	9 (7,10)	9 (6,12)	9 (7,12)	.16
Atypical clinical presentation, %	57/150 (38%)	89/819 (11%)	3/139 (2%)	.001
MMSE				
Final score, pts	7 (5,15)	13 (7,19)	18 (8,21)	.01
Longitudinal decline, pts lost per yr	-4.8 (-5.9,-2.1)	-1.4 (-3.4,-0.2)	-1.0 (-1.5,-0.23)	.001
**Neuropathologic findings**				
CorticoLimbic Index, pts	7.8 (5.9,9.2)	20 (16,25)	32 (31,33)	.001
Age at death, yr	72 (66,80)	81 (76,86)	86 (82,90)	.001
Brain weight, g	1040 (960,1140)	1040 (940,1140)	1040 (950,1120)	.40
Braak tangle stage	VI (V,VI)	VI (V,VI)	VI (V,VI)	.001
Thal amyloid phase	5 (5,5)	5 (5,5)	5 (5,5)	.68
Lewy body disease	25/175 (14%)	265/1014 (26%)	44/172 (26%)	.003
Kalaria cerebrovascular disease scale, pts	4 (2,5)	4 (2,6)	5 (2,6)	.004
TDP-43 positive, %	29/88 (33%)	168/305 (55%)	38/56 (68%)	.001

Data are medians (25th percentile to 75th percentile) unless noted. TDP-43 screening was performed in the amygdala corresponding to LATE-NC stage 1. AD=Alzheimer's disease. *APOE*=gene encoding apolipoprotein E. FLAME-AD=Florida Multiethnic Alzheimer's disease. CLix=CorticoLimbic index. g=grams. LATE-NC=limbic-predominant age-related TDP-43 encephalopathy neuropathologic change. MMSE=Mini-Mental State Examination. TDP-43=transactive response DNA binding protein of 43 kDa. yr=years.

© 2024 Kouri N et al. *JAMA Neurology*.

eTable 2. Clinicopathologic characteristics of the neuroimaging group

Characteristic	Alzheimer's disease neuropathologic subtypes (n=93)			*P* value
	**Hippocampal sparing** (n=18, CLix <10)	**Typical** (n=63, CLix ≥10 to <30)	**Limbic predominant** (n=12, CLix ≥30)	
Female, %	5/18 (28%)	21/63 (33%)	5/12 (42%)	.71
Education, yr	16 (12,18)	16 (13,18)	14 (12,16)	.27
APOE ε4 carriership, %	10/17 (59%)	33/60 (55%)	7/12 (58%)	1.00
**Clinical findings**				
Age at onset of cognitive symptoms, yr	54 (52,59)	70 (63,80)	77 (70,82)	**<.001**
Disease duration, yr	6.6 (5.8,8.1)	9.1 (5.8,11)	8.6 (5.7,11)	.45
Atypical clinical syndrome, %	9/18 (50%)	8/63 (13%)	1/12 (8%)	**.003**
Atypical clinical syndrome with dysexecutive AD, %	16/18 (89%)	15/63 (24%)	1/12 (8%)	**< .001**
MMSE longitudinal decline, pts lost per yr	-5.1 (-6.5,-3.8)	-1.3 (-2.8,-0.4)	-1.1 (-1.4,-0.7)	**< .001**
**Neuroimaging findings**				
Age at MRI, yr	62 (59,66)	80 (72,87)	85 (82,89)	**<.001**
Hippocampal volume	-0.90 (-1.4,-0.50)	-1.7 (-2.3,-1.0)	-2.2 (-2.5,-1.6)	**.002**
Age at Tau PET, yr	64 (62,66)	76 (71,87)	88 (84,90)	**.02**
Tau-PET parietal SUVr	2.6 (2.2,2.8)	1.3 (1.2,1.4)	1.3 (1.2,1.3)	**.04**
Tau-PET posterior cingulate and cuneus SUVr	2.6 (2.2,2.9)	1.4 (1.2,1.4)	1.3 (1.2,1.3)	**.02**
**Neuropathologic findings**				
CorticoLimbic Index, pts	6.9 (4.6,7.6)	21 (16,25)	33 (32,36)	**<.001**
Age at death, yr	64 (60,67)	81 (73,88)	87 (83,90)	**<.001**
Brain weight, g	1200 (1077,1280)	1310 (1153,1404)	1200 (1161,1360)	.07
Braak tangle stage	VI (VI,VI)	V (V,VI)	V (V,VI)	**.004**
Thal amyloid phase	5 (5,5)	5 (4,5)	5 (4,5)	.08
Kalaria cerebrovascular disease scale, pts	1.5 (0.0,3.0)	3.0 (3.0,6.0)	5.0 (3.0,7.0)	**.02**
TDP-43 positive, %	2/15 (13%)	16/57 (28%)	5/10 (50%)	.16
LATE-NC stage	0 (0,0)	0 (0,0)	0.5 (0,1.5)	**.01**

Data are medians (25th percentile to 75th percentile) unless noted. TDP-43 screening was performed in the amygdala corresponding to LATE-NC stage 1. *APOE*=gene encoding apolipoprotein E. g=grams. LATE-NC=limbic-predominant age-related TDP-43 encephalopathy neuropathologic change. MMSE=Mini-Mental State Examination. TDP-43=transactive response DNA binding protein of 43 kDa. yr=years.

© 2024 Kouri N et al. *JAMA Neurology*.

eTable 3. Clinicopathologic characteristics of the digital pathology subgroup

Characteristic	Alzheimer's disease neuropathologic subtypes (n=60)			*P* value
	**Hippocampal sparing** (n=20, CLix <10)	**Typical** (n=20, CLix ≥10 to <30)	**Limbic predominant** (n=20, CLix ≥30)	
Female, %	5/20 (25%)	9/20 (45%)	17/20 (85%)	.001
Education, yr	16 (14,18)	16 (14,18)	16 (12,16)	.71
APOE ε4 carriership, %	12/20 (60%)	14/20 (70%)	16/20 (80%)	.39
**Clinical findings**				
Age at onset of cognitive symptoms, yr	65 (58,68)	72 (65,76)	81 (78,87)	.001
Disease duration, yr	7.9 (6.3,10)	10 (7.3,15)	6.8 (6.0,8.6)	.18
Atypical clinical presentation, %	7/18 (39%)	5/20 (25%)	1/11 (9%)	.20
MMSE longitudinal decline, pts lost per yr	-5.6 (-5.6,-5.2)	-0.5 (-1.3,0.2)	-1.1 (-1.2,-0.8)	.03
**Neuropathologic findings**				
CorticoLimbic Index, pts	5.4 (4.4,5.8)	20 (19,21)	33 (32,35)	.001
Age at death, yr	71 (68,74)	82 (76,85)	87 (84,91)	.001
Brain weight, g	1050 (990,1130)	980 (910,1125)	1100 (1000,1130)	.15
Braak tangle stage	VI (V,VI)	VI (V,VI)	V (IV,VI)	.01
Thal amyloid phase	5 (5,5)	5 (5,5)	5 (5,5)	.87
Kalaria cerebrovascular disease scale, pts	4.0 (3.0,5.0)	4.0 (2.0,5.0)	4.0 (2.0,5.0)	.82
TDP-43 positive, %	6/20 (30%)	14/20 (70%)	18/20 (90%)	.001
LATE-NC stage	0 (0, 1.0)	1.5 (0, 2.0)	2.0 (1.0, 2.0)	.005

Data are medians (25th percentile to 75th percentile) unless noted. TDP-43 screening was performed in the amygdala corresponding to LATE-NC stage 1. The MMSE longitudinal decline was only available for a subset of the digital pathology subgroup (3 hippocampal sparing, 7 typical, 3 limbic predominant). Lewy body disease was not included as part of the digital pathology subgroup exclusion criteria. *APOE*=gene encoding apolipoprotein E. g=grams. LATE-NC=limbic-predominant age-related TDP-43 encephalopathy neuropathologic change. MMSE=Mini-Mental State Examination. TDP-43=transactive response DNA binding protein of 43 kDa. yr=years.

© 2024 Kouri N et al. *JAMA Neurology*.

eTable 4. Reference percentiles and thioflavin-S positive tangle counts for AD subtype classifier algorithm

Algorithm input	Label	Percentile	Neurofibrillary tangle count	
			Discovery	FLAME-AD
Hippocampal data				
CA1	H1	50th	12	12
Subiculum	H2	50th	20	20
(H1 + H2) / 2	H3	50th	17.5	16.5
Association cortex data				
Superior Temporal	C1	50th	10.5	10
Inferior Parietal	C2	50th	8	7
Middle Frontal	C3	50th	5	5
(C1 + C2 + C3) / 3	C4	50th	8.7	8
Corticolimbic ratio				
H3 / C4	R	25th	1.11	1.09
		75th	3.60	3.75

The discovery series was used to originally define the AD subtype algorithm (n=889).[3] In the current study, the Florida Autopsied Multi-Ethnic Alzheimer's disease (FLAME-AD) series was used as the updated reference population (n=1361) for the development of the corticolimbic index (CLix). H = Hippocampus, C = Cortex, R = Ratio between the hippocampus and cortex.

© 2024 Kouri N et al. *JAMA Neurology*.

eTable 5. Characteristics of primary antibodies used in this study

Antibody information						Digital Pathology
**Clone**	**Target**	**Epitope**	**Manufacturer**	**Catalog #**	**Dilution**	**Macro**
AT8	Hyperphosphorylated tau	pS202, pT205	Thermo Scientific	MN1020	1:2500	CD
GT-38[11]	AD-specific tau conformer	Conformational	Abcam USA	ab246808	1:2500	PPC
6F/3D	Amyloid-β	8-17aa of Aβ	DAKO	M0872	1:100	PPC
CD68 (KP1)	Activated microglia/ macrophages	N/A	Agilent Technologies (DAKO)	M081401-2	1:750	CD
GFAP (GA-5)	Astrogliosis	N/A	Biogenex	MU020-UC	1:5000	PPC
MC2085[12]	TDP-43 C-terminal	VFIPKPFR	Petrucelli lab (Mayo Clinic)	N/A	1:3000	---
phospho-TDP-43 (11-9)	pTDP-43	CMDSKS(p)S(p)GWGM	Cosmo Bio USA	CAC-TIP-PTD-M01A	1:5000	---

All antibodies were mouse IgG1. For each antibody, the same antigen retrieval protocol was used: 30 minutes steam in dH_2O, except 6F/3D, which was first treated in 98% formic acid followed by 30 minutes steam in dH_2O. CD=color-deconvolution. PPC=positive-pixel count.

© 2024 Kouri N et al. *JAMA Neurology*.

eTable 6. Slides stained and evaluated for the digital pathology subgroup

	Diagnostic slides		Immunohistochemical slides for digital pathology					Total
	H&E	Thioflavin-S	AT8 tau	GT-38 tau	6F/3D amyloid-β	GFAP	CD68	
**Hippocampal sparing AD**	20	20	20	20	20	20	20	140
**Typical AD**	20	20	20	20	20	20	20	140
**Limbic predominant AD**	20	20	20	20	20	20	20	140
**All**							**420 x 4 tissue blocks**	**= 1680 slides**

Four tissue blocks included one posterior hippocampus at level of lateral geniculate (CA1, subiculum) and three association cortices (superior temporal, inferior parietal, and middle frontal). H&E=hematoxylin and eosin, AT8=hyperphosphorylated tau marker (phosphorylated Ser202 and Thr205), GT-38=AD-specific tau conformer marker (conformational epitope), GFAP=glial fibrillary acidic protein (marker for astrogliosis), CD68=cluster of differentiation 68 (marker for activated microglia/macrophages).

© 2024 Kouri N et al. *JAMA Neurology*.

eTable 7. CLix distribution among AD subtypes stratified by ethnoracial status

	Alzheimer's disease neuropathologic subtypes in FLAME-AD (n=1361)				*P* value
	**Hippocampal sparing** (CLix <10)	**Typical** (CLix ≥10 to <30)	**Limbic predominant** (CLix ≥30)	**Total (n=1358)**	
**Black/African American decedents**					
n (%)	2 (14%)	8 (57%)	4 (29%)	14	---
CLix score	7.6 (6.5,8.7)	22 (18,26)	33 (33,33)	25 (18,32)	**.006**
**Hispanic/Latin American decedents**					
n (%)	4 (6%)	52 (84%)	6 (10%)	62	---
CLix score	7.9 (6.8,8.7)	19.7 (16,25)	32 (31,33)	20 (16,26)	**.001**
**Non-Hispanic White decedents**					
n (%)	169 (13%)	951 (74%)	162 (13%)	1282	---
CLix score	7.8 (5.9,9.2)	20 (16,25)	32 (31,34)	20 (14,27)	**.001**
**All decedents**					
n (%)	175 (13%)	1011 (74%)	172 (13%)	1358	**---**
CLix score	7.8 (5.9,9.2)	20 (16,25)	32 (31,33)	20 (14,27)	**.001**

Medians (1st quartile to 3rd quartile) and counts (%) are reported, and *P* values result from Kruskal-Wallis rank sum test for continuous variables. Two Native American decedents (CLix=18, 22) and one Asian decedent (CLix=22) from FLAME-AD were not included in the analysis due to the small sample size. AD=Alzheimer's disease. CLix=CorticoLimbic index. FLAME-AD=Florida Autopsied Multi-thnic Alzheimer's disease.

© 2024 Kouri N et al. *JAMA Neurology*.

eTable 8. Regional neuropathologic measures in the AD digital pathology subgroup

Variables	Alzheimer's disease neuropathologic subtypes				*P* value		
	Hippocampal sparing (n=20, CLix <10)	Typical (n=20, CLix ≥10 to <30)	Limbic predominant (n=20, CLix ≥30)	Overall	HpSp vs. Typical	HpSp vs. Limbic	Typical vs. Limbic
**Thioflavin-S tangle counts**							
CA1	5.0 (3.8,7.0)	16 (12,31)	20 (15,26)	**.001**	**.001**	**.001**	.53
Subiculum	10 (5.0,10)	30 (15,55)	35 (27,40)	**.001**	**.001**	**.001**	.76
Superior temporal ctx.	17 (12,25)	17 (8.8,24)	6.0 (3.8,8.0)	**.001**	.68	**.001**	**.001**
Inferior parietal ctx.	19 (15,24)	12 (4.8,18)	2.0 (1.0,3.2)	**.001**	**.04**	**.001**	**.001**
Middle frontal ctx.	15 (10,21)	7.0 (3.0,13)	1.0 (0.00,2.0)	**.001**	**.003**	**.001**	**.001**
**Hyperphosphorylated tau (AT8 burden, %)**							
CA1	22 (17,28)	26 (15,36)	26 (20,40)	.24	.33	.11	.45
Subiculum	38 (26,41)	33 (18,43)	38 (30,49)	.40	.64	.36	.21
Superior temporal ctx.	32 (23,38)	29 (14,38)	16 (10,27)	**.006**	.22	**.001**	.06
Inferior parietal ctx.	32 (23,42)	23 (10,28)	6.7 (3.3,12)	**.001**	**.003**	**.001**	**.001**
Middle frontal ctx.	30 (22,38)	19 (8.3,26)	1.9 (0.90,5.2)	**.001**	**.002**	**.001**	**.001**
**AD-specific tau conformer (GT-38 burden, %)**							
CA1	1.1 (0.90,1.4)	3.4 (1.6,5.9)	4.1 (2.7,5.3)	**.001**	**.001**	**.001**	.49
Subiculum	1.5 (0.90,2.1)	3.6 (1.7,8.5)	4.6 (3.2,7.1)	**.001**	**.004**	**.001**	.46
Superior temporal ctx.	1.7 (1.0,2.2)	2.5 (0.60,5.4)	0.70 (0.40,1.1)	**.009**	.64	**.002**	**.03**
Inferior parietal ctx.	4.6 (2.5,7.1)	3.0 (1.0,4.8)	0.70 (0.40,1.0)	**.001**	.06	**.001**	**.002**
Middle frontal ctx.	2.1 (1.8,3.8)	1.5 (0.60,3.4)	0.30 (0.20,0.40)	**.001**	.10	**.001**	**.003**
**Amyloid-β (6F/3D burden, %)**							
CA1	0.30 (0.30,0.60)	0.40 (0.20,1.4)	0.80 (0.50,1.3)	**.03**	.57	**.002**	.17
Subiculum	0.40 (0.30,0.60)	0.80 (0.50,1.4)	1.2 (0.80,1.5)	**.001**	**.04**	**.001**	.31
Superior temporal ctx.	2.6 (2.0,3.2)	3.1 (1.4,4.4)	2.3 (1.3,2.9)	.23	.90	.10	.20
Inferior parietal ctx.	4.1 (3.3,5.5)	3.6 (3.0,4.7)	2.9 (1.9,3.9)	.08	.36	**.02**	.22
Middle frontal ctx.	3.6 (1.9,5.9)	3.0 (2.6,5.6)	2.9 (2.0,3.9)	.47	.97	.27	.34
**Astrogliosis (GFAP burden, %)**							
CA1	13 (8.5,18)	33 (22,43)	30 (28,40)	**.001**	**.001**	**.001**	.95
Subiculum	16 (12,20)	33 (27,42)	34 (27,42)	**.001**	**.001**	**.001**	.97
Superior temporal ctx.	22 (18,29)	29 (20,41)	21 (18,29)	.08	.07	.95	**.04**
Inferior parietal ctx.	32 (23,40)	28 (24,33)	20 (18,24)	**.001**	.58	**.001**	**.001**
Middle frontal ctx.	27 (20,35)	29 (24,40)	18 (13,30)	**.02**	.57	**.03**	**.007**
**Activated microglia/macrophages (CD68 burden, %)**							
CA1	0.54 (0.39,0.79)	1.2 (0.96,1.8)	1.3(0.94,1.5)	**.001**	**.001**	**.001**	.90
Subiculum	0.46 (0.35,0.70)	1.2 (0.96,1.5)	1.2 (1.0,1.4)	**.001**	**.001**	**.001**	.82
Superior temporal ctx.	0.30 (0.21,0.43)	0.59 (0.44,1.0)	0.38 (0.23,0.47)	**.003**	**.002**	.51	**.004**
Inferior parietal ctx.	0.46 (0.32,0.75)	0.75 (0.51,0.98)	0.40 (0.32,0.57)	**.02**	.06	.37	**.004**
Middle frontal ctx.	0.35 (0.25,0.58)	0.63 (0.48,0.88)	0.39 (0.29,0.48)	**.02**	**.01**	.68	**.01**

© 2024 Kouri N et al. *JAMA Neurology*.

Variables	Alzheimer's disease neuropathologic subtypes			Overall	*P* value		
	Hippocampal sparing (n=20, CLix <10)	Typical (n=20, CLix ≥10 to <30)	Limbic predominant (n=20, CLix ≥30)		HpSp vs. Typical	HpSp vs. Limbic	Typical vs. Limbic

Medians (1st quartile to 3rd quartile). P values for overall difference among three groups resulted from Kruskal-Wallis rank-sum test; p values for pairwise comparison resulted from Wilcoxon rank-sum test. AD=Alzheimer's disease. Ctx=cortex. Limbic=Limbic predominant AD.

eFigures

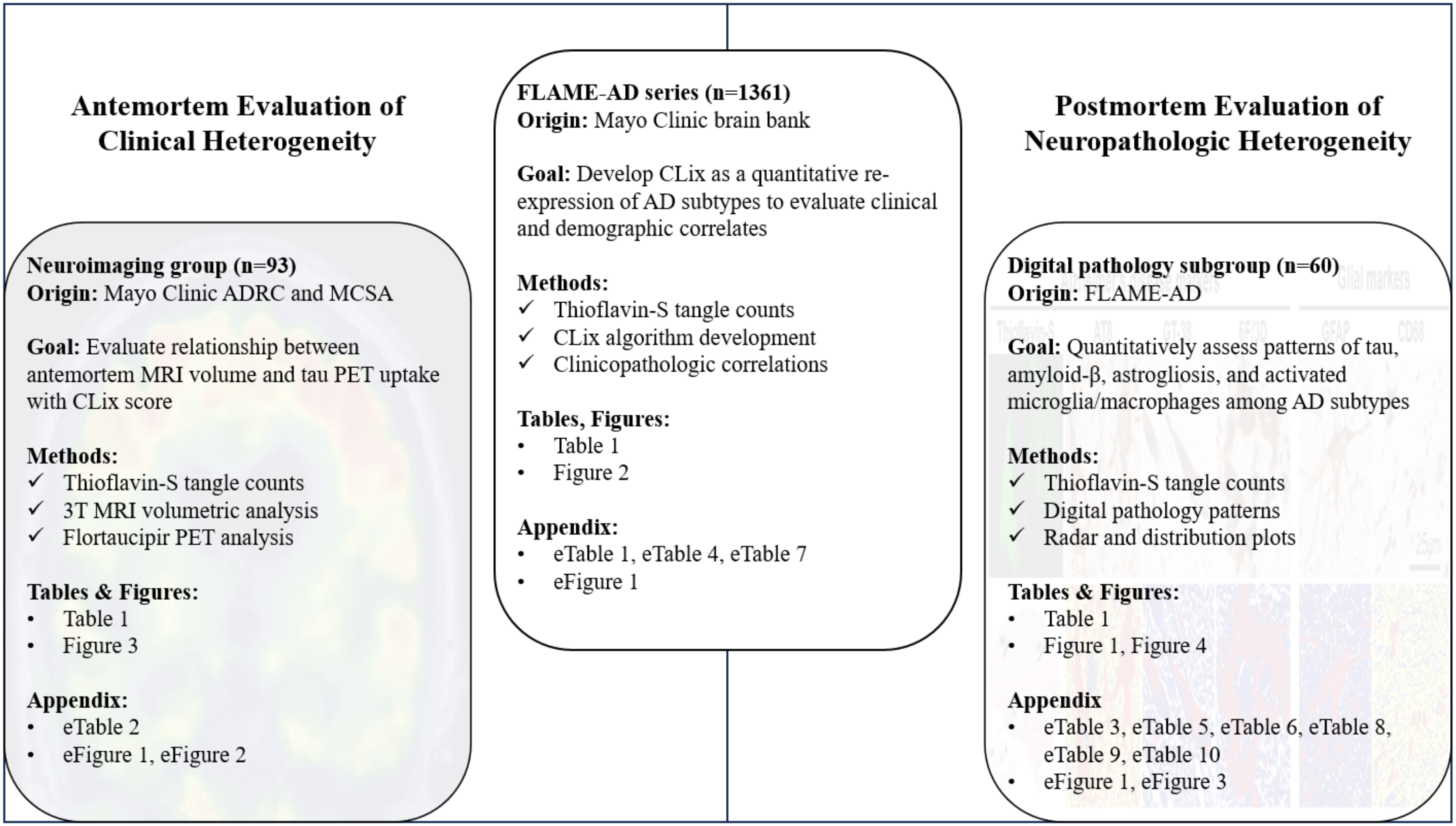

© 2024 Kouri N et al. *JAMA Neurology*.

eFigure 1. Overview of three study groups evaluated with CLix

Antemortem evaluation of clinical heterogeneity was evaluated in the FLAME-AD series (middle) and neuroimaging group (left), whereas postmortem evaluation of neuropathologic heterogeneity was evaluated in the FLAME-AD group (middle) and digital pathology subgroup (right). Pertinent information regarding sample size, goal, methodologies applied, tables, figures, and appendix reference are ;included for each of the three groups analyzed. Abbreviations: AD, Alzheimer's disease; ADRC, Alzheimer's Disease Research Center; CLix, CorticoLimbic index; FLAME-AD, Florida Multiethnic Alzheimer's disease; MCSA, Mayo Clinic Study of Aging; MRI, magnetic resonance imaging; PET, positron emission tomography.

© 2024 Kouri N et al. *JAMA Neurology*.

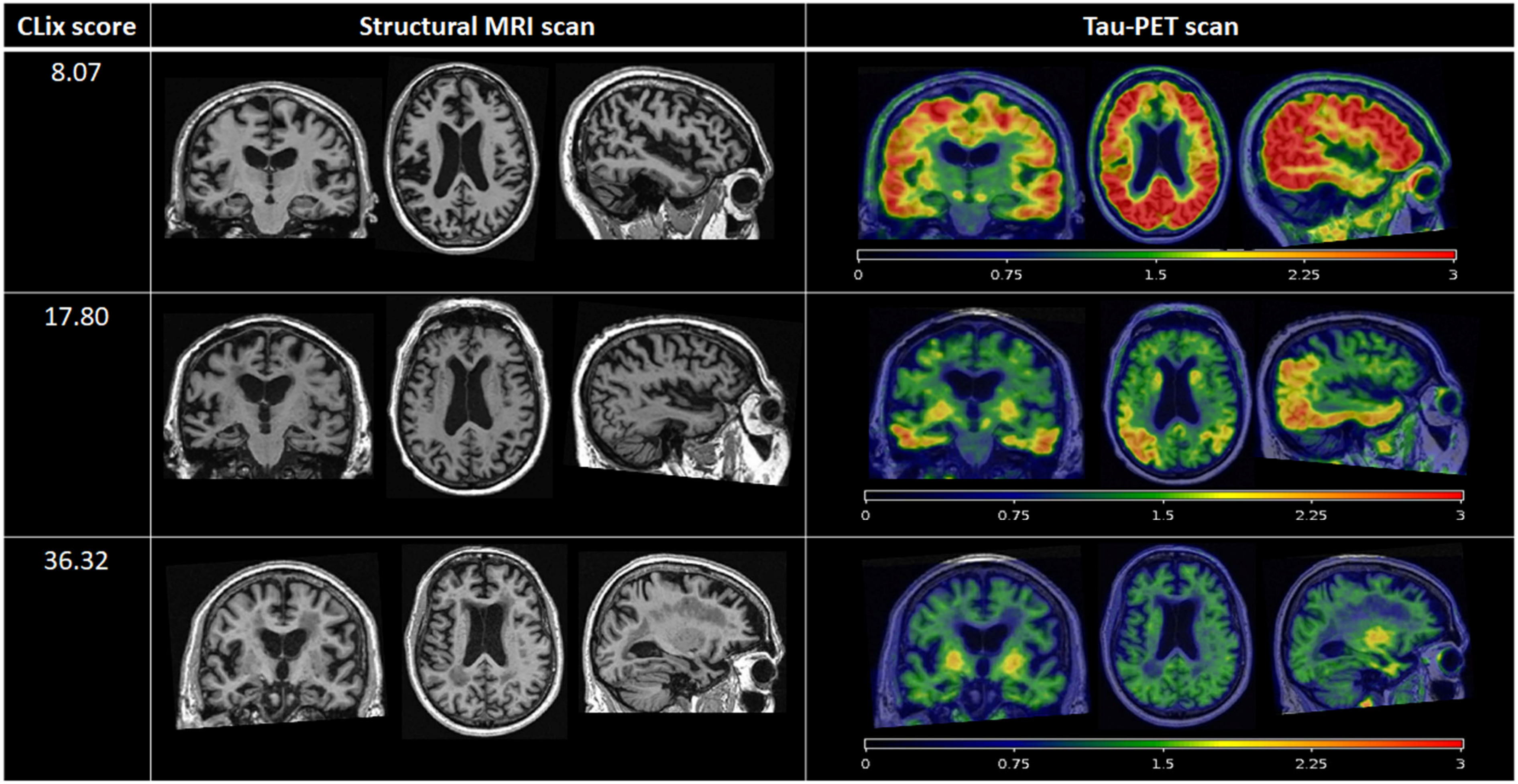

eFigure 2. Structural MRI and tau-PET scans across CLix scores

CLix score is a spatial score representative of corticolimbic tangle involvement that extends well to structural MRI (left) and tau-PET (right). Example cases from the neuroimaging group were all neuropathologically diagnosed with high likelihood AD. (Top) Low CLix score corresponded to relatively preserved limbic structures with extensive cortical tau-PET uptake as shown by representative images in a 61-year-old man imaged 2.0 years prior to death. (Middle) A moderate CLix score corresponded to affected limbic structures with medial temporal lobe and cortical tau-PET uptake as shown by representative images in an 86-year-old man imaged 0.8 years prior to death. (Bottom) High CLix score corresponded to greatly affected limbic structures with minimal cortical tau-PET uptake as shown by representative images in an 89-year-old man imaged 1.6 years prior to death. Abbreviations: AD, Alzheimer's disease; CLix, CorticoLimbic index; MRI, magnetic resonance imaging; PET, positron emission tomography. Note: The color bar values indicate the value of the T-statistic with higher tracer uptake shown in warmer colors. Arrow on tau PET map points to posterior cingulate and cuneus region.

© 2024 Kouri N et al. *JAMA Neurology*.

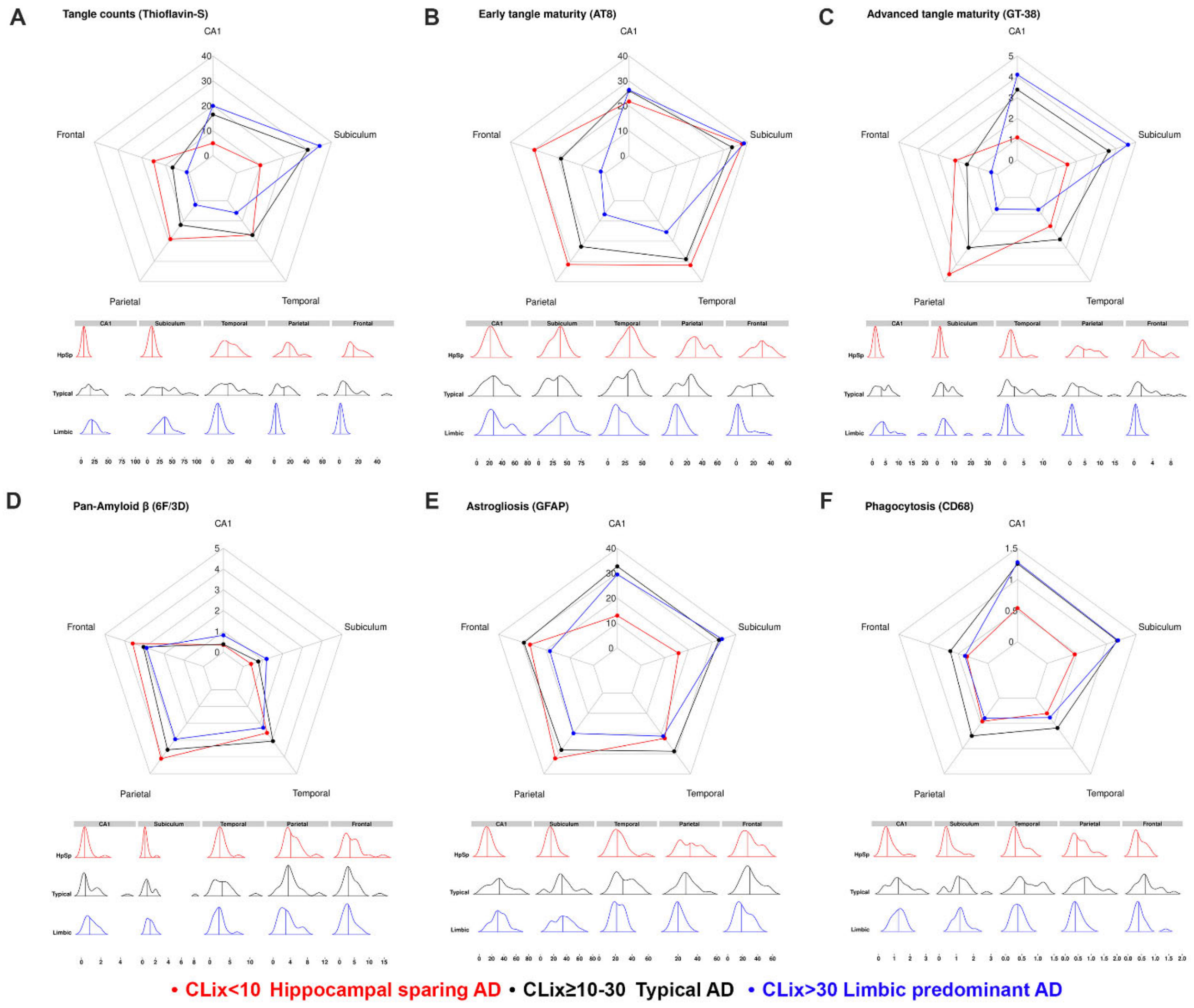

eFigure 3. Regional quantitative measures and distributions in the digital pathology subgroup among AD neuropathologic subtypes

Radar plots and distribution plots comparing hippocampal sparing (red), typical (black), and limbic predominant AD (blue) across five brain regions for thioflavin-S-positive tangles (A), hyperphosphorylated tau marker with AT8 (B), AD-specific tau conformer with GT-38 (C), pan-Aβ with 6F/3D (D), activated microglia/macrophages with CD68 (E), and astrogliosis with GFAP (F). Red signifies CLix <10 (hippocampal sparing AD), black signifies CLix ≥10 to <30 (typical AD), and blue signifies CLix ≥30 (limbic predominant AD).

© 2024 Kouri N et al. *JAMA Neurology*.

eReferences

1. Santos OA, Pedraza O, Lucas JA, et al. Ethnoracial differences in Alzheimer's disease from the FLorida Autopsied Multi-Ethnic (FLAME) cohort. *Alzheimer's & Dementia*. 2019/05/01/ 2019;15(5):635-643. doi:https://doi.org/10.1016/j.jalz.2018.12.013
2. Liesinger AM, Graff-Radford NR, Duara R, et al. Sex and age interact to determine clinicopathologic differences in Alzheimer's disease. *Acta neuropathologica*. Sep 15 2018;doi:10.1007/s00401-018-1908-x
3. Murray ME, Graff-Radford NR, Ross OA, Petersen RC, Duara R, Dickson DW. Neuropathologically defined subtypes of Alzheimer's disease with distinct clinical characteristics: a retrospective study. *Lancet Neurol*. Sep 2011;10(9):785-96. doi:10.1016/s1474-4422(11)70156-9
4. Murray ME, Cannon A, Graff-Radford NR, et al. Differential clinicopathologic and genetic features of late-onset amnestic dementias. *Acta neuropathologica*. Sep 2014;128(3):411-21. doi:10.1007/s00401-014-1302-2
5. Braak H, Braak E. Neuropathological stageing of Alzheimer-related changes. *Acta neuropathologica*. 1991;82(4):239-59.
6. Graff-Radford J, Yong KXX, Apostolova LG, et al. New insights into atypical Alzheimer's disease in the era of biomarkers. *Lancet Neurol*. Mar 2021;20(3):222-234. doi:10.1016/S1474-4422(20)30440-3
7. Thal DR, Rub U, Orantes M, Braak H. Phases of A beta-deposition in the human brain and its relevance for the development of AD. *Neurology*. Jun 25 2002;58(12):1791-800. doi:10.1212/wnl.58.12.1791
8. Nelson PT, Lee EB, Cykowski MD, et al. LATE-NC staging in routine neuropathologic diagnosis: an update. *Acta neuropathologica*. Feb 2023;145(2):159-173. doi:10.1007/s00401-022-02524-2
9. Deramecourt V, Slade JY, Oakley AE, et al. Staging and natural history of cerebrovascular pathology in dementia. *Neurology*. 2012;78(14):1043-1050. doi:10.1212/WNL.0b013e31824e8e7f
10. Nguyen AT, Kouri N, Labuzan SA, et al. Neuropathologic scales of cerebrovascular disease associated with diffusion changes on MRI. *Acta neuropathologica*. Dec 2022;144(6):1117-1125. doi:10.1007/s00401-022-02465-w
11. Gibbons GS, Banks RA, Kim B, et al. Detection of Alzheimer Disease (AD)-Specific Tau Pathology in AD and NonAD Tauopathies by Immunohistochemistry With Novel Conformation-Selective Tau Antibodies. *J Neuropathol Exp Neurol*. Mar 1 2018;77(3):216-228. doi:10.1093/jnen/nly010
12. Zhang YJ, Xu YF, Cook C, et al. Aberrant cleavage of TDP-43 enhances aggregation and cellular toxicity. *Proc Natl Acad Sci U S A*. May 5 2009;106(18):7607-12. doi:10.1073/pnas.0900688106
13. Murray ME, Przybelski SA, Lesnick TG, et al. Early Alzheimer's Disease Neuropathology Detected by Proton MR Spectroscopy. *The Journal of Neuroscience*. 2014;34(49):16247. doi:10.1523/JNEUROSCI.2027-14.2014
14. Chang CC, Chow CC, Tellier LC, Vattikuti S, Purcell SM, Lee JJ. Second-generation PLINK: rising to the challenge of larger and richer datasets. *Gigascience*. 2015;4:7. doi:10.1186/s13742-015-0047-8
15. Wang K, Li M, Hakonarson H. ANNOVAR: functional annotation of genetic variants from high-throughput sequencing data. *Nucleic Acids Res*. Sep 2010;38(16):e164. doi:10.1093/nar/gkq603
16. Roberts RO, Geda YE, Knopman DS, et al. The Mayo Clinic Study of Aging: design and sampling, participation, baseline measures and sample characteristics. *Neuroepidemiology*. 2008;30(1):58-69. doi:10.1159/000115751
17. Rocca WA, Yawn BP, St Sauver JL, Grossardt BR, Melton LJ, 3rd. History of the Rochester Epidemiology Project: half a century of medical records linkage in a US population. *Mayo Clinic proceedings*. Dec 2012;87(12):1202-13. doi:10.1016/j.mayocp.2012.08.012
18. St Sauver JL, Grossardt BR, Yawn BP, et al. Data resource profile: the Rochester Epidemiology Project (REP) medical records-linkage system. *Int J Epidemiol*. Dec 2012;41(6):1614-24. doi:10.1093/ije/dys195
19. Petersen RC, Roberts RO, Knopman DS, et al. Prevalence of mild cognitive impairment is higher in men. The Mayo Clinic Study of Aging. *Neurology*. Sep 7 2010;75(10):889-97.

© 2024 Kouri N et al. *JAMA Neurology*.

20. Townley RA, Graff-Radford J, Mantyh WG, et al. Progressive dysexecutive syndrome due to Alzheimer's disease: a description of 55 cases and comparison to other phenotypes. *Brain Commun*. 2020;2(1):fcaa068. doi:10.1093/braincomms/fcaa068
21. Schwarz CG, Gunter JL, Wiste HJ, et al. A large-scale comparison of cortical thickness and volume methods for measuring Alzheimer's disease severity. *Neuroimage Clin*. 2016;11:802-812. doi:10.1016/j.nicl.2016.05.017
22. Schwarz CG, Therneau TM, Weigand SD, et al. Selecting software pipelines for change in flortaucipir SUVR: Balancing repeatability and group separation. *Neuroimage*. Sep 2021;238:118259. doi:10.1016/j.neuroimage.2021.118259
23. Ashburner J, Friston KJ. Voxel-based morphometry--the methods. *Neuroimage*. Jun 2000;11(6 Pt 1):805-21. doi:10.1006/nimg.2000.0582
24. Mirra SS, Heyman A, McKeel D, et al. The Consortium to Establish a Registry for Alzheimer's Disease (CERAD): Part II. Standardization of the neuropathologic assessment of Alzheimer's disease. *Neurology*. 1991;41(4):479-479.
25. Montine TJ, Phelps CH, Beach TG, et al. National Institute on Aging-Alzheimer's Association guidelines for the neuropathologic assessment of Alzheimer's disease: a practical approach. *Acta neuropathologica*. Jan 2012;123(1):1-11. doi:10.1007/s00401-011-0910-3
26. Ossenkoppele R, Pijnenburg YA, Perry DC, et al. The behavioural/dysexecutive variant of Alzheimer's disease: clinical, neuroimaging and pathological features. *Brain*. Sep 2015;138(Pt 9):2732-49. doi:10.1093/brain/awv191

© 2024 Kouri N et al. *JAMA Neurology*.
