# Supplementary material for: Clinicopathologic Heterogeneity and Glial Activation Patterns in Alzheimer Disease
Source: JAMA Neurol. 2024 Apr 15;81(6):619–29. doi: 10.1001/jamaneurol.2024.0784 (PMC11019448; doi:10.1001/jamaneurol.2024.0784)
Supplement: Supplement 2. — Data Sharing Statement. [file jamaneurol-e240784-s002.pdf]

Data Sharing Statement

Kouri. Clinicopathologic Heterogeneity and Glial Activation Patterns in Alzheimer Disease. *JAMA Neurol*. Published April 15, 2024. doi:10.1001/jamaneurol.2024.0784

Data
Data available: No

Additional Information
Explanation for why data not available: We have attached a Data Sharing Statement as a supplemental file. We are also pasting the statement below: The Corticolimbic Index creation algorithm and imputation for missing tangle counts values were described in detail and deposited on GitHub (https://github.com/Translational-Neuropathology-Lab). All requests for de-identified raw and analyzed data will be reviewed by Mayo Clinic's Legal Department and Mayo Clinic Ventures to verify whether a request is subject to intellectual property or confidentiality obligations. Requests for patient-related data not included in the paper will not be considered. Any de-identified data and materials that can be shared will be released via a Data Use/Share Agreement or Material Transfer Agreement. Neuroimaging data applications can be submitted via Data Sharing and Resources - Alzheimer's Disease Research Center - Mayo Clinic Research.
